# Robustly Repeatable, Permeable, and Multi‐Axially Stretchable, Adhesive Bioelectronics With Super‐adaptive Conductive Suction Cups for Continuously Deformable Biosurfaces

**DOI:** 10.1002/advs.202500346

**Published:** 2025-03-31

**Authors:** Gyun Ro Kang, Gui Won Hwang, Dohyun Lim, Seung Hwan Jeon, Minwoo Song, Chan‐Hwa Hong, Hye Jin Kim, Changhyun Pang

**Affiliations:** ^1^ School of Chemical Engineering Sungkyunkwan University (SKKU) 2066 Seobu‐ro, Jangan‐gu Suwon Gyeonggi‐do 16419 Republic of Korea; ^2^ Mechanical Metrology Group Korea Research Institute of Standards and Science Daejeon 34113 Republic of Korea; ^3^ Electronics and Telecommunications Research Institute (ETRI) Daejeon 34129 Republic of Korea; ^4^ Samsung Advanced Institute for Health Sciences and Technology (SAIHST) Sungkyunkwan University Suwon Gyeonggi‐do 16419 Republic of Korea

**Keywords:** bioadhesive, bioelectronics, composite, kirigami, wet adhesion

## Abstract

Skin‐integrated wearable bioelectronics offer immense potential for continuous health monitoring, diagnosis, and personalized therapy. However, robustly repeatable and permeable adhesive interfaces with omnidirectional stretchability for adaptability to continuously deforming skin surface remain a critical challenge and often results in issues such as delamination, void, and signal degradation. This study presents a highly adaptable bioelectronic device with a repeatable, robust and biocompatible adhesive interfaces designed for dynamic wet skin surfaces. The device integrates a conductive softened‐double‐layered octopus‐inspired nanocomposites adhesive and kirigami metastructure (cs‐OIA_k). The cs‐OIA_k achieves skin‐like softness, electrical stability (*ΔR/R_0_* < 10, under 10 000 cycles) and omnidirectional stretchability (a maximum of 200%) to accommodate skin deformation. Additionally, the hierarchical structural design of cs‐OIA_k enables repeatable robust adhesion (> 10 000 cycles) and vertical alignment to ensure reversible adhesion against dynamically deforming surface (−30% to 100%, depending on skin thickness, site, and age) without skin irritation. Based on these characteristics, the highly adaptable skin‐adhesive bioelectronics are demonstrated to achieve reliable electrocardiogram (ECG) and electromyogram (EMG) signal measurements even under shoulder movements with extreme skin deformation. This approach utilizing multi‐axially stretchable, repeatable robust adhesives, permeable and biocompatible bioelectronics provides new insights for the development of advanced wearable systems and human–machine interfaces.

## Graphical abstract

It is presented bioelectronics that exhibit exceptional adaptability to dynamic skin surfaces through an integrated structural and material design. These omnidirectionally stretchable, skin‐compliant, mechanically robust, and permeably biocompatible kirigami‐based devices minimize skin irritation and conductivity loss and offer new insights for the development of advanced wearable systems and human–machine interfaces.

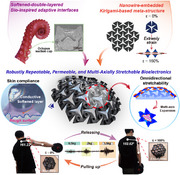

## 1 Introduction

Skin‐integrated bioelectronics offer transformative healthcare applications, including high‐fidelity physiological monitoring, real‐time health status analysis, and personalized therapy.^[^ ^1^, ^2^, ^3^, ^4^, ^5^ ^]^ For effective skin integration, it is critical to ensure optimal adaptability to a dynamic skin surface, including multi‐directional stretchability, permeability and repeatable robust skin adhesive interfaces. These issues are even more difficult to achieve in the skin that has low elasticity (< 500 kPa) and high deformation (−30% to 100%, depending on skin thickness, site, and age) in various directions and constantly undergoes various movements.^[^ ^6^ ^]^ Materials engineering, the use of nanocomposites, is a widely adopted approach to enhance skin adaptability while maintaining biocompatibility, skin‐like softness, deformability, conformal skin contact, and reliable sensing performance.^[^ ^5^, ^7^, ^8^, ^9^, ^10^, ^11^, ^12^, ^13^, ^14^ ^]^ However, achieving stretchability in bioelectronic materials presents inherent challenges because of the contradictory requirements of mechanical durability, electrical conductivity, and deformability. As the material strain, its susceptibility to issues such as strain localization, cracking, and degradation of the conductive network increases, all of which can impair its performance. Furthermore, bioelectronic devices often struggle to accommodate the constantly shifting and dynamic nature of the skin surface, which leads to challenges such as shifting contact points, degraded electrical performance, and detachment or formation of voids. These limitations underscore the urgent need for innovative bioelectronic solutions offering multi‐axial stretchability, robust repeatability adhesion, biocompatibility with breathable to maintain reliable functionality under the complex and ever‐changing conditions of skin deformation.

Diverse strategies with geometrical structures or stretchable materials have been developed to retain the contact location and electrical properties under high strain on non‐flat complicated skin surfaces.^[^ ^15^, ^16^, ^17^ ^]^ Promising approaches include liquid metals and advanced conductive polymers like Poly(3,4‐ethylenedioxythiophene) polystyrene sulfonate (PEDOT:PSS), which offer high deformability and electrical repeatability under strain.^[^ ^9^, ^18^, ^19^, ^20^, ^21^, ^22^ ^]^ However, in environments where moisture such as skin occurs and permeability to air and water is required, it loses electrical performance and easily delamination or irritate the skin, so there are limitations to direct application without a separated encapsulation processing. Also, serpentine electrodes undergo lateral stretching, but are often limited in long‐term, dynamically continuously moving skin applications due to electrical degradation and unstable skin contact when subjected to high strains in multi‐axis directions simultaneously.^[^ ^23^, ^24^ ^]^ Kirigami and origami designs enable localized stretchability, introducing enhanced adaptability under complex deformation. However, conventional devices that rely on chemical adhesives for skin attachment have significant limitations, including rapid delamination in dynamic and wet environments, limited repeatability, low permeability and high skin irritation. Therefore, the development of bioelectronic systems necessitates reliable adhesive interfaces that demonstrate repeatability, biocompatibility, and robustness to address these challenges in skin applications. In this regard, recent structural and material approaches to bioelectrode technologies are summarized in **Table** **1** for their performance and functionality (adhesion performance, stretchability, repeatability, and skin irritation index).

**Table 1:** Comparison of various stretchable adhesive bioelectronics. Statistical significance was calculated: *p < 0.05; **p < 0.01; ***p < 0.001; ****p < 0.0001.

Material	Structural Design	Maximum strain [%]	Repeating cycle [#] [Number of experiment]		Adhesion performance [p‐value]	Skin irritation index	Refs.
			Tensile strain	Adhesion			
1D‐2D Carbon/polydimethylsiloxane (PDMS) nanocomposite	N/A	100	N/A	30 (1)	Dry ∼ 13 kPa (‐)	N/A	[25]
					N/A		
Multi‐walled carbon nanotube (MWCNT) electrode on PDMS	N/A	30	2500 (1)	100 (10)	Dry ∼ 27 kPa (‐) Wet ∼ 20 kPa (‐)	N/A	[29]
Carbon black (CB)/PDMS nanocomposite	N/A	60	10 000 (1)	1000 (10)	Dry ∼ 50 kPa (‐) Wet ∼ 20 kPa (‐)	No irritation(‐)	[48]
Ag–Au nanocomposite	Serpentine	50	N/A	N/A	Chemical adhesive	N/A	[12]
Liquid Metal composite	Kirigami structure (biaxial)	105	100 (1)	N/A	Chemical adhesive	N/A	[17]
Aramid nanofiber(ANF‐PVA)	Kirigami membrane	120	5000 (1)	N/A	Chemical adhesive	N/A	[15]
Carbon nano tube (CNT)‐Ag flakes/Poly‐urethane (PU) nanocomposite	Hexagonal mesh‐pattern	150	1000 (1)	N/A	Dry ∼ 15 kPa (‐)	N/A	[36]
					Wet ∼ 12 kPa (‐)		
Single‐walled carbon nano tube (SWCNT)/Ecoflex nanocomposite	Kirigami structure (triaxial)	200	10 000 (1)	10 000 (1)	Dry ∼ 36 kPa (****)	No irritation (ΔI/I < 0.1)	Our work
					Wet ∼ 72 kPa (****)		

Inspired by natural adhesive structures, bio‐inspired interface designs such as octopus suction cups and diving beetle feet have been explored as potential solutions for skin‐interface biosensors.^[^ ^25^, ^26^, ^27^, ^28^, ^29^ ^]^ These systems demonstrate remarkable adaptability to soft, rough, wet, vulnerable surfaces due to their ability to maintain stable adhesion under complex conditions.^[^ ^30^, ^31^, ^32^ ^]^ However, their high modulus limits compliance and omni‐directional strain, leading to unstable signal acquisition on highly dynamic skin surfaces. Efforts to mimic the microstructure of adhesive biostructures such as the soft infundibulum and acetabulum of the octopus suction cup have therefore demonstrated enhanced adaptability to dynamic surfaces.^[^ ^33^ ^]^ Despite these advancements, such designs frequently low electrical conductivity, confining their application to auxiliary adhesives rather than fully integrated sensor platforms. Achieving both high conductivity and skin‐compatible multidirectional stretchability within these bio‐inspired systems remains a critical challenge. For practical applications to skin‐interface biosensors, innovative solutions are needed that perfectly satisfy the conflicting requirements of conductivity, multidirectional stretchability, and repeatable robust adhesive interfaces with permeability.

This study introduces a bioelectronic platform featuring exceptional repeatable robust adhesion and multidirectional stretchability to adapt to dynamic dry and wet skin conditions. This is achieved using a conductive softened‐double‐layered octopus‐inspired adhesive (cs‐OIA) combined with kirigami metastructure (cs‐OIA_k). The kirigami structures provide skin‐like softness, high stretchability (≈200%), and stable conductivity by minimizing material deformation and preserving the percolation network, which facilitates the flow of electricity through carbon nanotubes. The kirigami structure imparts skin‐like softness, high elasticity (≈200%), and stable conductivity by minimizing material deformation and preserving the percolating network, which facilitates the flow of electricity through carbon nanotubes. The cs‐OIA ensures robust, reversible adhesion in both dry (≈37 kPa) and wet (≈72 kPa) environments, conforming to rough and curved skin. The integrated cs‐OIA_k distributes stress evenly across its kirigami backbone, while the octopus‐inspired adhesive maintains conformal contact even on 90° curved surfaces, preventing wrinkles and folds for stable adhesion under shear and peel forces. This design yields high elasticity and electromechanical stability, allowing components like light emitting diodes (LEDs) to function reliably under multi‐directional strain. Demonstrations show the device effectively capturing electrocardiogram (ECG) and electromyogram (EMG) signals without noise, even on highly deformed skin (≈−30% to 100% strain). This permeable, conductive adhesive bioelectronics with reliable, repeatable robust adhesive interfaces minimizes skin irritation and offers a promising new approach for advanced wearable systems and human‐machine interfaces. The following section presents detailed results and discussions, while key technical terms are summarized **Table** **2** in the experimental section.

**Table 2:** Specific explanations and definitions of scientific terms.

Term	Definition
Infundibulum	A funnel‐shaped structure, biologically referring to the beginning or widening of a specific tube or organ.
Acetabulum	A part of a suction cup observed in the adhesive structures of arthropods or cephalopods, which generates negative pressure through elastic deformation to exert strong adhesive force.
Kirigami	A technique of cutting and folding paper or thin materials to create new shapes, a structural pattern design technique that increases omnidirectional elasticity and reduces stress applied to the surface.
Percolation network	A network structure in which conductive particles are interconnected to form a path through which electricity can flow.
Compliance	It indicates the degree to which an object deforms when force is applied and can be considered the inverse of stiffness.
Conductivity	It measures how easily a substance can transfer electricity or heat.
Modulus	A physical property that indicates the degree of resistance when a material is deformed, and Young's modulus is generally included as an example.
Positive Poisson's ratio	A material property that indicates that the width decreases in the orthogonal direction as the length increases when an object is stretched and is observed in most common materials.
Negative Poisson's ratio	A material property where the material expands in all directions when stretched, providing exceptional flexibility and durability.
Electrocardiogram	A technology that records the electrical activity of the heart to measure periodic signals and is one of the important biosignal detection methods.
Electromyogram	A biosignal detection technology that detects the electrical activity of muscles to measure muscle movement and tension.
Signal to noise ratio	It indicates the ratio of the intensity of a useful signal to the intensity of noise and is used to evaluate the performance of electronic devices and signal processing.

## 2 Results and Discussion

### 2.1 Conductive Softened‐Double Layered Octopus‐Inspired Adhesive with Kirigami Structure

**Figure 1:**
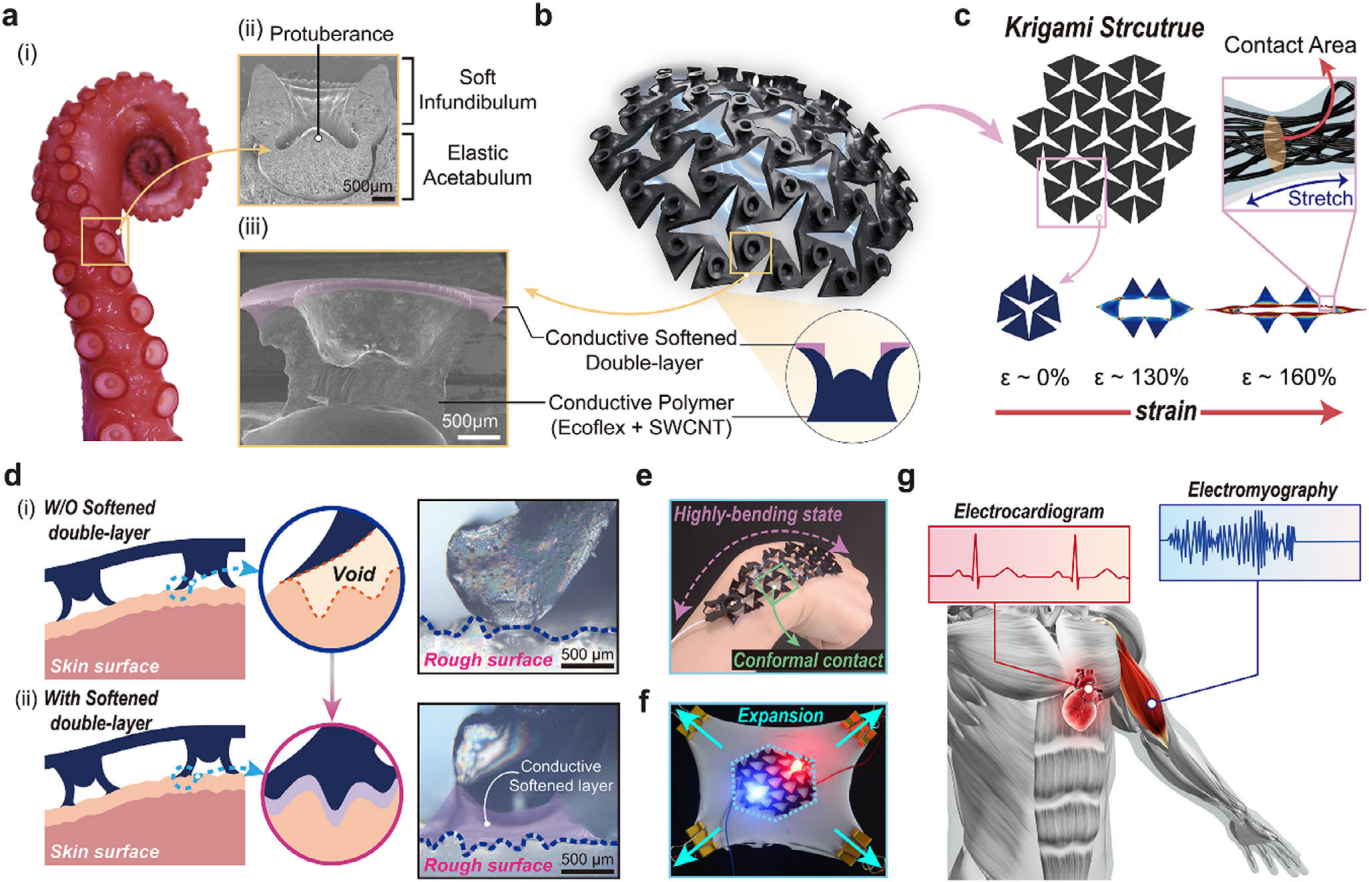
Highly adaptive conductive softened‐double layered, octopus‐inspired, robustly repeatable adhesive electronics with omni‐stretchable kirigami‐based auxetic structure for bio‐signal monitoring under dynamically moving skin conditions. a) Arm of *Octopus vulgaris* with suckers for compliant adhesion. i) Photographic image of an Octopus vulgaris tentacle. ii) SEM cross‐section image of an octopus suction cup with anatomical details. iii) SEM cross‐section image of the cs‐OIA adhesive. b) Illustration of the conductive softened‐double layered octopus‐inspired adhesive architecture (cs‐OIA_k) electrode patch. c) Kirigami‐based auxetic meta‐structure under tensile strain (up to ≈160%). The figure inset illustrates the stretchable composite containing a 3D percolated network of conductive fillers embedded in an elastomer matrix. d) Cross‐sectional profiles at the skin interface of the suction cups i) without and ii) with conductive softened double layer. Optical microscopy images of the OIA and cs‐OIA. e) Photographic image showing the conformal contact property of the cs‐OIA_k to the skin under wrist extension. f) Photograph of stretchable cs‐OIA_k with interconnected LEDs on the substrate under multi‐axial strain. g) Schematic of the cs‐OIA_k adhesive bioelectronics to measure ECG and EMG signals.

A conductive softened‐double‐layered octopus‐inspired adhesive patch with a kirigami pattern (cs‐OIA_k) was designed to enhance the adaptability through multidirectional stretchability and repeatable, robust adhesive interfaces with permeability, for wearable bioelectronics on dynamic biological surfaces. (**Figure** **1**a–c). As shown in Figure 1a‐i,ii, the tentacles of *Octopus vulgaris* contain numerous suction cups with a soft infundibulum and an elastic acetabulum to enable attachment to various surfaces.^[^ ^34^, ^35^ ^]^ Interestingly, it was discovered that the robust adhesion strength of the octopus suction cups was because of a negative pressure induced by the structural deformation of the acetabulum. Furthermore, a soft infundibulum and the appropriate deformation of tentacles play a crucial role in maintaining a sustained attachment with exceptional adaptability to dynamic and rough bio‐surfaces. Inspired by these structural and material properties, a conductive softened double‐layered octopus‐inspired architecture (cs‐OIA) was designed to achieve a structural deformation‐based negative pressure mechanism and adaptability to rough bio‐surfaces (Figure 1a‐iii). Additionally, cs‐OIA integrated with a kirigami‐based meta‐structure enabled independent movement of each suction cup, similar to an octopus tentacle (Figure 1b,c; Figure S3, Supporting Information). These structural features increase the omni‐stretchability, reduce the stress applied to the surface, and minimize damage to the electrically conductive nanocomposite matrix. As shown in Figure 1d, the bioelectronics in this study were designed by integrating kirigami patterns for stress dissipation and a diameter of 3 mm and height of 2 mm flared suction cup with a conductive softened double layer (Figure S4, Supporting Information). The device incorporated with these adhesion structures achieved conformal contact with rough skin surfaces (Figure 1d), which demonstrates the effectiveness of the conductive softened double layer. This layer enhanced the skin conformance capacity of the device, thereby improving its adhesive stability and ensuring optimal contact with an irregular skin surface. Furthermore, the characteristics of the kirigami pattern facilitated robust adhesion to the surface in cs‐OIA_k while maintaining stable electrical performance as an electrode under multi‐axial deformation (Figure 1e,f). As shown in Figure 1e, the cs‐OIA_k device attached to the wrist maintained stable adhesion even under continuously extreme body movements. In addition, a consistent electrical property under multi‐axial strain was demonstrated by the LED connected to the cs‐OIA_k electrode, which continued to illuminate uniformly even when the substrate was subjected to multi‐directional tension (Figure 1f). It was confirmed that, through an integrated approach, cs‐OIA_k provided stable and versatile bio‐signal monitoring even on dynamic skin surfaces (Figure 1g). The cs‐OIA_k bioelectronics maintained high adaptability on both sweaty and rough skin and effectively detected ECG and EMG signals even on highly deformable skin (for example, shoulder and wrist). In addition, Table 1 provides a comparative analysis of various stretchable adhesive bioelectronics, detailing their structural design, maximum strain, cyclic tensile durability, adhesive performance, and skin compatibility. The results, supported by statistical significance, demonstrate the superior dry and wet adhesion stress, along with remarkable mechanical stability, of the single‐walled carbon nano tube (SWCNT)/Ecoflex kirigami‐structured nanocomposite developed in this study. Our design exhibits superior electrical stability, enhanced biocompatibility, and extended reusability, while achieving exceptional adhesion performance in both dry and wet environments.

### 2.2 Electrical and Mechanical Stability Against Multi‐Axial Tensile Strain

**Figure 2:**
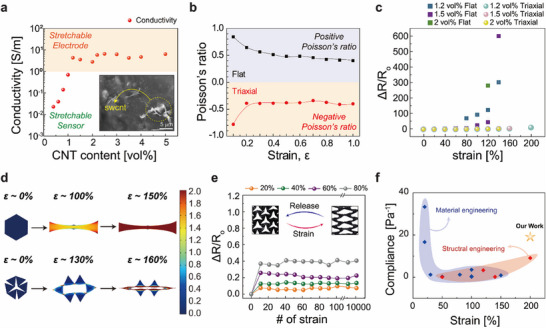
Mechanical and electrical characteristics of the SWCNT composites fabricated for multi‐axial stretchable electric device. a) Conductivity of the SWCNTs/Ecoflex nanocomposite according to the volume fraction of CNTs. The sorting tracks (1 Sm^−1^) to the stretchable electrode and sensor are shown in the graph. b) Poisson's ratios of the auxetic and non‐auxetic structures. c) The electrical properties of the cs‐OIA were represented by the rate of resistance change with applied strain (≈200%), according to the composite concentration and structure. Error bars represent standard deviations (n = 5) d) FEM simulation of volumetric change under tensile strain, which is the main element in the electrical signal stability of auxetic structure. e) Reproducible electrical signals of the 1.2 vol% triaxial structure under 10 000 stretching (each strain %) cycles. f) Comparison of the maximum strain and compliance between material,^[^ ^7^, ^9^, ^10^, ^11^, ^12^, ^13^, ^25^, ^26^, ^29^, ^31^ ^]^ and structural engineering,^[^ ^7^, ^15^, ^16^, ^17^ ^]^ for sustained electrode performance.

To enhance the adaptability to skin surface, omni‐stretchable and contractible conductors were designed using a kirigami‐structured single‐walled carbon nanotube (SWCNT) composite material, using a material‐structural synergetic approach (**Figure** **2**). Initially, the conductivity of the SWCNT/Ecoflex nanocomposites was measured based on the conductive filler (SWCNT) volume fraction in the polymer matrix (Ecoflex) (Figure 2a). Therefore, composite materials with different SWCNT volume fractions were prepared using the same fabrication process. The conductivity of the SWCNT/Ecoflex nanocomposites increased with increasing SWCNT volume fractions and reached ≈10 S m^−1^ at contents above 1.2 vol%. This electrical property was attributed to the additional conduction paths created by the enhanced physical contact and tunneling effects between the SWCNT particles. The optimal conductivity for stretchable electrode applications should be greater than 1 S m^−1^, which appears above a minimum of 1.2 vol% SWCNT content.^[^ ^36^, ^37^, ^38^ ^]^ Based on these findings, the mechanical properties of three different vol. % SWCNT/Ecoflex nanocomposites (1.2, 1.5, and 2.0 vol%) suitable for tensile electrodes were investigated (Figure S5, Supporting Information). The stress–strain (S–S) curves measured using a custom device showed that the modulus increased with increasing SWCNT content (Figure S6, Supporting Information). This indicates that although the conductivity improves with increasing SWCNT content, adding highly elastic fillers to the soft polymer matrix reduces the softness and stretchability, which potentially limits skin adaptability. Therefore, to enhance the omni‐stretchability of materials incorporating conductive fillers with high elastic modulus, a kirigami‐based triaxial structure was implemented (Figure 2b–f). As shown in Figure 2b, the conventional flat structure shows a positive Poisson's ratio, which indicates that the material shrinks in the other axial direction when stretched. In contrast, the triaxial structure shows a negative Poisson's ratio as the structural deformation is preferential, which indicates that the material expands in the other axial direction when stretched, which facilitates the simultaneous lateral expansion under tensile strain (Figure 2b). The negative Poisson's ratio generated by the structural deformation enhanced the stretchability and ensured excellent electrical stability by maintaining a consistent conduction path, even under strain. As shown in Figure 2c and Figure S7 (Supporting Information), the kirigami‐based triaxial composite electrode exhibited significantly enhanced electrical stability under tensile strain compared to the flat structure, irrespective of the SWCNT content (maximum ΔR/R_0_: kirigami, ≈10%; flat, ≈600%). Moreover, finite element method (FEM) simulations indicate that under tension, the material strain in the flat structure increased rapidly. In contrast, the kirigami structure primarily underwent structural deformation through 3D geometric reorganization before material deformation (Figure 2d). Under tensile strain, both structural and material deformations occurred, and effective strain owing to material deformation was an important factor that affected the conductivity. The effective strain that affected the conductivity from a point in time after complete structural rearrangement is described by Equation. (1):

(1) $$\varepsilon_{esc}=\varepsilon_{ms}\;-\frac{\nu }{2}\left(\varepsilon_{ms}+\varepsilon_{ms}^{2}\right)$$

where ε_*esc*_ is the effective strain influencing the electrical conductivity, ε_*ms*_ is the material strain ratio, and ν is the material‐specific Poisson's ratio (see Supporting Information for a detailed description).

The effective strain influencing the electrical conductivity of the 3D kirigami‐structured electrodes derived from this theory was surprisingly similar to the experimental results (Figure S8, Supporting Information). Furthermore, the material strain calculated according to this theory was confirmed by the theoretical and experimental data of the material strain under tension, based on FEM simulations (Figure S8, Supporting Information). Specifically, the kirigami‐based electrode maintained very similar initial states, whereas the flat structure exhibited a drastic change in the spacing between the SWCNT particles, even at moderate tension. Finally, due to the unique stress distribution characteristics of the kirigami structure, material fatigue was minimized, and the electrical stability and consistency of the 1.2 vol% triaxial structure were confirmed over 10 000 cycles at various tensile strains (20%, 40%, 60%, and 80%) (Figure 2e; Figures S9, S10, Supporting Information). This study compares the stretchability and compliance of bioelectronic systems utilizing existing materials and structural engineering, with a focus on the electrically stable range (*ΔR/R_0_* < 10) (Figure 2f). Compliance, defined as the reciprocal of the elastic modulus, indicates the material's capacity for deformation and adaptability. While previous research has emphasized improving compliance or stretchability through materials or design independently, our approach integrates both aspects. The outcome is an advanced bioelectronic system exhibiting exceptional stretchability and compliance. This synergistic approach not only expands the potential applications of these devices but also establishes a new standard for high‐performance, stretchable bioelectronics.

### 2.3 Adhesion Characteristics of the cs‐OIA_k

**Figure 3:**
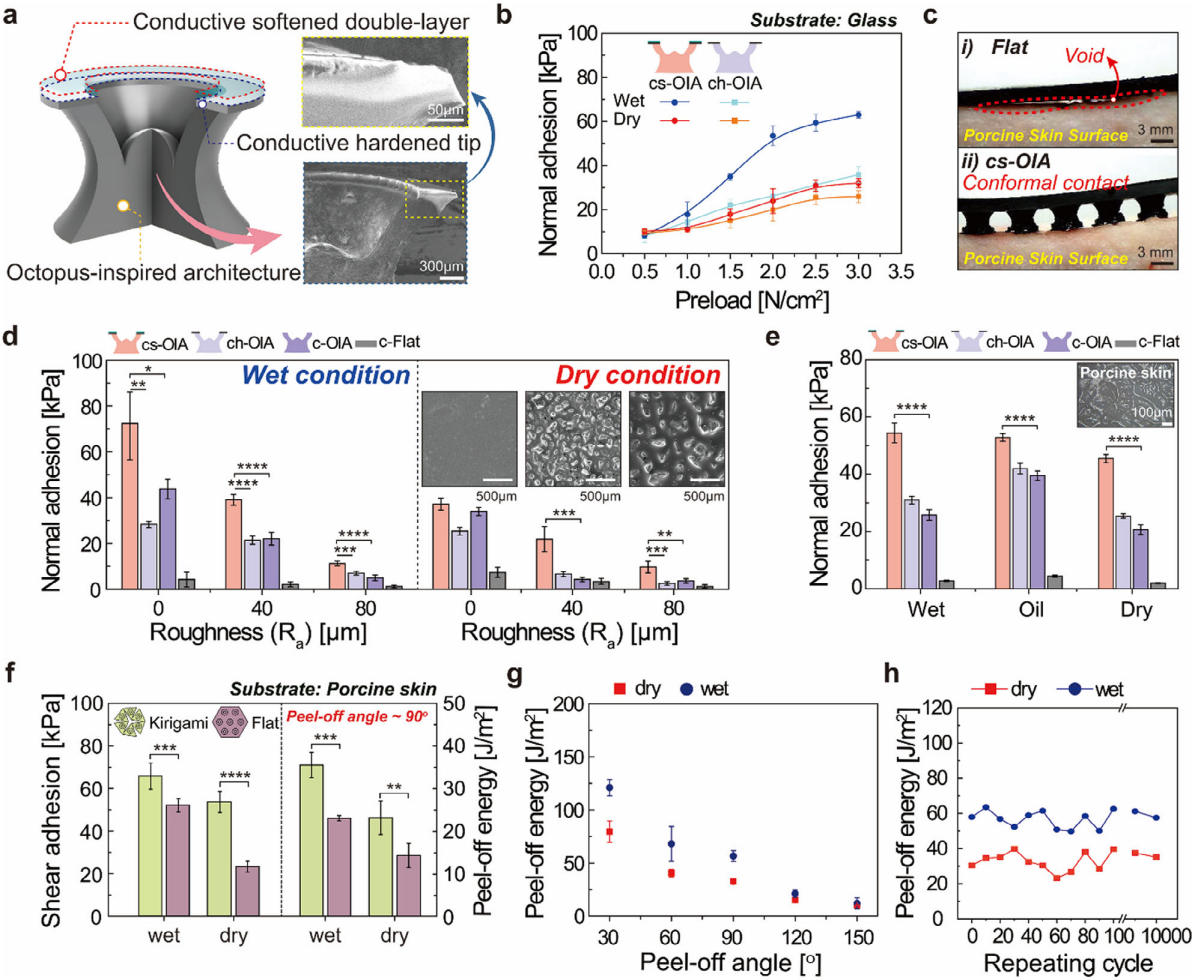
Adhesion performance of the bioinspired adhesive architecture with auxetic pattern on various types of surfaces under dry and wet conditions. a) Schematic of cs‐OIA. SEM cross‐sectional images of conductive‐softened double‐layered adhesive architecture. b) Normal adhesion stress with varying preload. Error bars represent standard deviations (n = 5) c) Optical microscopy (OM) images of super‐adaptively conformal contact on a porcine skin surface i) without and ii) with cs‐OIA. d, e) Normal adhesion of cs‐OIA, ch‐OIA, c‐OIA, and c‐flat on dry/wet surfaces with varying roughness (Ra = 0, 40 80 µm) and porcine skin. Error bars represent standard deviations (n = 5) f) Shear and peel‐off adhesion properties of the adhesives with or without the kirigami structure as the backbone. Error bars represent standard deviations (n = 5) g) Adhesion properties with different peeling angles (30°, 60°, 90°, 120°, and 150°) of the cs‐OIA_k. Error bars represent standard deviations (n = 5) h) Repeatability and durability of the cs‐OIA_k (≈10 000 cycles). Statistical significance was calculated: *p < 0.05; **p < 0.01; ***p < 0.001; ****p < 0.0001.

A cs‐OIA structure coated with a conductive soft elastomer suitable for dynamic biological surfaces was developed through material and structural optimization (**Figure** **3**a–e). It comprised SWCNT/Ecoflex nanocomposites and SWCNT/soft elastomers, which are biomaterials suitable for cytotoxicity, immunogenicity, and biocompatibility (Figure 3a).^[^ ^12^, ^39^, ^40^ ^]^ To ensure consistent electrical performance of the cs‐OIA structure, the conductivities of both the suction cup and softened double layer were designed to be within the same range (Figure S11, Supporting Information). The adhesion mechanism of the fabricated cs‐OIA consists of three stages: 1) Contact stage: Contact with the adhesion target; 2) Preload stage: Structural deformation owing to the applied preload; 3) Preload removal stage: Structural deformation because of the material elasticity and the change in the internal cavity volume as the applied preload is removed, which forms a robust negative pressure‐based adhesion.^[^ ^41^, ^42^, ^43^ ^]^ The theoretical total wet adhesion was calculated using Equation. (2):

(2) $$\begin{array}{cc} \sigma_{t,wet} & =[\left(1-\gamma_{w}\right)\frac{P_{0}\pi {{D^{\prime \prime }}_{in}}^{2}}{4}+\frac{\pi \cdot \left({{D^{\prime \prime }}_{out}}^{2}-{{D^{\prime \prime }}_{in}}^{2}+4r^{2}\right)}{4}\\ & \;\cdot \gamma \left(\frac{\cos\vartheta_{1}+\cos\vartheta_{2}}{h}\right)+l\cdot \gamma ]\;\cdot n \end{array}$$

where *γ_w_* is a compensation factor with a value between 0 and 1 owing to seal leakage at the cs‐OIA regions of contact, *P*_0_ is the atmospheric pressure (≈101.3 kPa), *D*_in_^′′^ is the inner diameter of the contact surface when vacuum is established in the inner chamber, *D*_out_^′′^ is the outer diameter of the contact surface when vacuum is established in the inner chamber, *r* is the radius of the dome in contact with the substrate, *γ* is the surface tension of water, *θ_1_* and *θ_2_* are the contact angles on the adhesive and engaged substrate, respectively, *h* is the liquid gap, and *l* is the outer circumference. Additionally, the adhesion force under dry conditions can be predicted mainly by the van der Waals force and the volume‐change‐dependent suction stress (see Figures S1 and S2, Supporting Information for a detailed description).

The adhesion stress was measured in various directions (normal, shear, and peel‐off) on different substrates using custom‐built adhesion equipment (Figure S12, Supporting Information for further details). For normal adhesion, the adhesion stress was measured by gradually increasing the preload (≈3.0 N cm^2^) under both dry and wet conditions (Figure 3b). Under both conditions, the adhesion stress of both the cs‐OIA (dry (7.96–35.81 kPa) and wet (8.28–69.03 kPa)) and the conductive hardened tip octopus‐inspired architecture (ch‐OIA) (dry ≈9.44–25.89 kPa) and wet (10.29–32.04 kPa)), increased with increasing preload (0.5–3.0 N cm^2^), and a saturation trend was observed in the 2.0–3.0 N cm^2^ range. Overall, these results confirm that exceptional adhesion occurs when a conductively softened double layer is present. However, because the adhesive stress reached saturation within a similar preload range, regardless of the presence of the double layer, it is evident that the structural deformation of the adhesive plays a key role in generating negative pressure. Also, adhesion stress was found to be significantly higher in wet environments compared to dry conditions. This is attributed to the non‐expansibility of water, which allows for the formation of a more complete vacuum during the vacuum formation process, thereby enhancing adhesion strength (more detailed in Movie S1, Supporting Information). The conductive softened elastomer stamping process resulted in a softer surface (skin modulus of ≈1–100 kPa) for the adhesive cups, which improved their adaptability and compliance to the skin. As shown in Figure 3c, the conductive flat structure (c‐Flat) formed voids on the rough and curved porcine skin surfaces, whereas the cs‐OIA structure demonstrated excellent conformal contact, as verified by OM. The excellent adhesion performance on rough skin (10–50 µm) was validated through adhesion tests on various rough surfaces (Figure 3d).^[^ ^44^ ^]^ The adhesion strength decreased as the roughness increased; however, the cs‐OIA structure exhibited good adhesion (≈9–11 kPa) even at 80 µm. This was attributed to the softened double‐layered structure, which facilitated conformal contact with rough surfaces to maintain vacuum and featured a protuberance that induced vacuum by maximizing the volume change. Furthermore, the cs‐OIA structure exhibited robust adhesion under various porcine skin surface conditions (dry ≈45 kPa, extremely dry ≈37 kPa, cut ≈25 kPa, wet ≈54 kPa, and oily surface ≈53 kPa), which indicates its high adaptability to the diversity of human skin (Figure 3e; Figure S13, Supporting Information).

Subsequently, the backbone of the kirigami pattern was integrated with the adhesive structure optimized in Figure 2, and the effect of this combination was investigated (Figure 3f–h). In the cs‐OIA structure, the kirigami pattern significantly improved the adhesion performance and peel‐off energy in the shear and peel‐off directions compared with that of the flat pattern under dry/wet skin conditions (Figure 3f; Figure S14, Supporting Information). FEM simulations were used to investigate the effect of combining the kirigami‐patterned backbone and the adhesive structure (Movie S2, Supporting Information). Generally, during patch detachment, the stress on the adhesive cup is concentrated in the peeling direction, which causes weakened sealing and subsequent detachment by preventing additional crack propagation at adhesion interface. Therefore, the stress transmitted through the backbone initiated subsequent continuous detachment of the adhesive cup. In a flat structure, when a patch was removed, the stress of the backbone was immediately transferred to the successive adhesive cup, whereas in the kirigami pattern, the stress of the backbone was dispersed through node rotation, which alleviates the sequential detachment pattern. Furthermore, when the adhesion performance of the kirigami structure was measured based on the detachment angle affecting the peel‐off energy, the peel‐off energy increased as the angle decreased, which is in accordance with the Kendall theory (Figure 3g).^[^ ^45^ ^]^ Unlike the flat structure, the cs‐OIA structure integrated with the kirigami structure maintained stable adhesion and electrical performance even under extreme deformation conditions of the porcine skin (up to 150° bending) (Figure S15, Supporting Information). Moreover, the adhesive performance was maintained even after ≈10 000 cycles (Figure 3h). Although the adhesion decreased due to surface contamination after repeated use, the original adhesion performance was completely restored by simple surface cleaning, confirming that long‐term use is possible (Figure S16, Supporting Information). These comprehensive results validate the robust functionality of cs‐OIA across diverse biological surface conditions, directly supporting its practical applicability for human skin applications.

### 2.4 Adhesion Stability of the cs‐OIA_k Attached on the Multi‐Axially Extensible Surface

**Figure 4:**
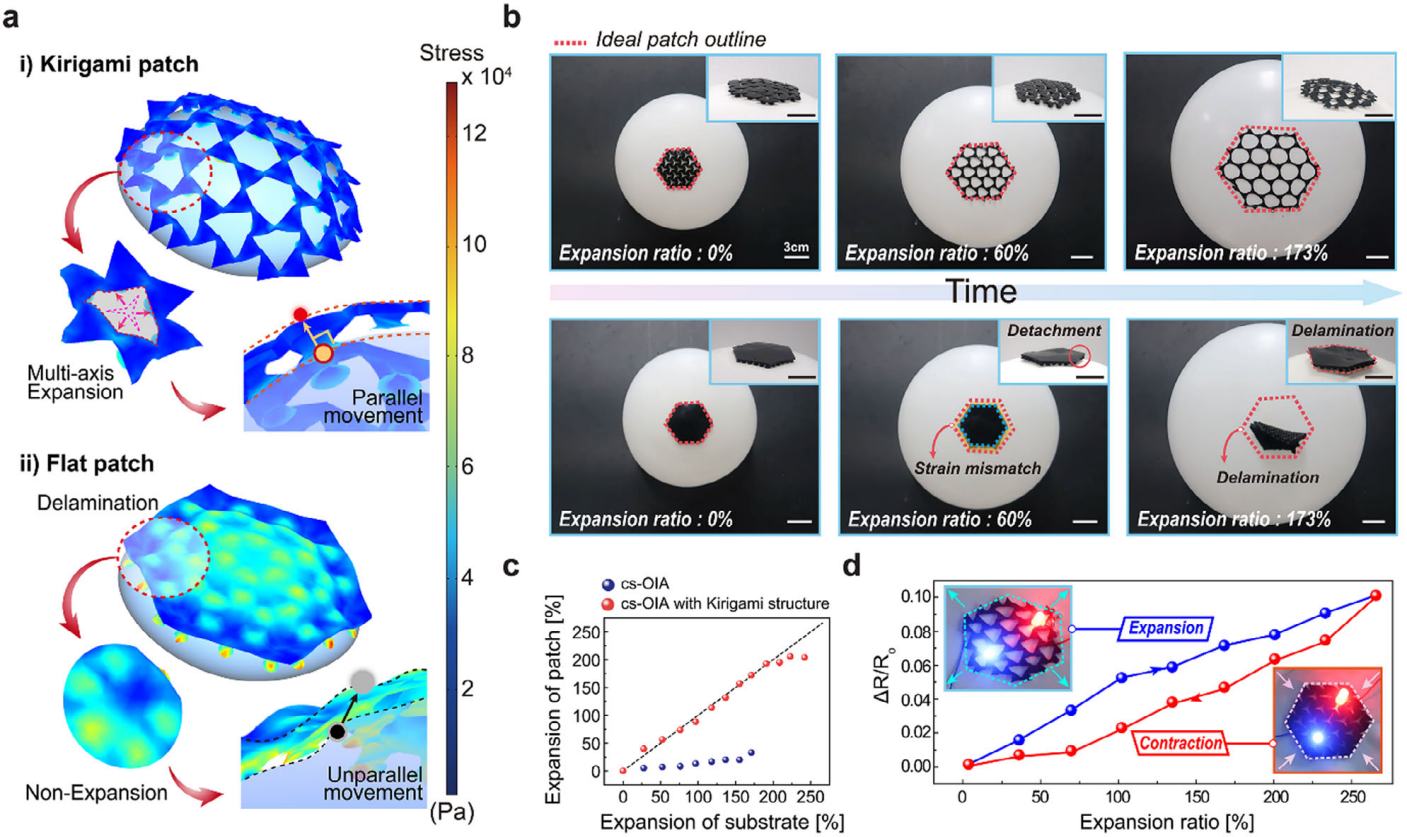
Robustly repeatable adhesion stability of the cs‐OIA_k attached on multi‐axial stretchable curved surface. a) FEM simulation of the stress distribution of kirigami‐based and flat adhesives. b) Snapshot of a kirigami‐based adhesive and flat adhesive on a curved balloon that expanded up to 173% over time. c) The strain of the two adhesive patches (kirigami and flat) based on the strain of the substrate d) The electrical properties of the 1.2 vol% kirigami‐based adhesive patch according to expansion. Inset image is the multi‐axis deformation image of a cs‐OIA based on a stretchable kirigami with interconnected LEDs on the substrate.

It was demonstrated that the kirigami‐patterned backbone enabled effective conformal contact without wrinkling or folding and maintaining stable electrical properties when placed on an inflating spherical surface (**Figure** **4**). As shown in the FEM simulation analysis (Figure 4a; Movie S3, Supporting Information), only a minimal stress concentration occurred within the kirigami structure when a strain of up to 173% was applied to the patch by the multi‐axially expanding curved surface. This enabled complete contact with the inflating spherical surface. To achieve effective adhesion on continuously moving and rough skin, the adhesive cup must remain vertically aligned on the curved surface to ensure proper sealing and suction strength. The excellent stress distribution of the kirigami pattern enabled the maintenance of the adhesive cup angle close to the vertical alignment, even with the increase in surface curvature, thereby ensuring optimal adhesive stress. In contrast, the flat structure failed to effectively disperse the stress, which resulted in concentrated stress and folding in the patch. This patch folding impeded effective adhesion to the curved surface as the adhesive cup angle deviated significantly from 90° with increasing curvature. The inadequate stress distribution of the flat pattern limited its surface conformance, which reduced its overall adhesion and sealing performance. Therefore, the Kirigami pattern was applied as the backbone of the electrode to improve its expandability by increasing the maximum elongation and reducing the stress applied to the surface, thereby reducing electrode damage.

Experiments were performed to validate the FEM simulation results. Initially, to demonstrate the adaptability of kirigami‐patterned and non‐patterned patches on an expanding substrate, the patches were affixed to a balloon that was subsequently inflated to a consistent volume over time (Figure 4b; Movie S4, Supporting Information). The backbone of the kirigami‐patterned patch stretched in all directions and evenly dispersed the stress, which helped maintain high adhesion (Figure S17, Supporting Information). This demonstrates their superior adaptability to object deformation. Accordingly, the cs‐OIA_k electrode maintained stable adhesion and electrical resistance while the balloon was expanded up to 173% and contracted down to −30%. In contrast, the high strain mismatch between the flat patch and the balloon caused the non‐patterned patch to detach from the balloon surface at an expansion ratio of ≈60% (Figure S18, Supporting Information). When the expansion ratio reached 173%, the flat patch completely delaminated from the balloon surface. This was expressed as the ratio of the expansion rate of the balloon surface to that of the patch, as shown in Figure 4c. The cs‐OIA structure with a Kirigami pattern enabled continuous powering of light‐emitting diodes (LEDs) without brightness changes, even with expansion and contraction, owing to its high stretchability, high electrical conductivity, and electromechanical stability (Figure 4d; Movie S5, Supporting Information).

### 2.5 Biosignal Monitoring with Long‐Term Stability and Biocompatibility Under Various Environmental Conditions

**Figure 5:**
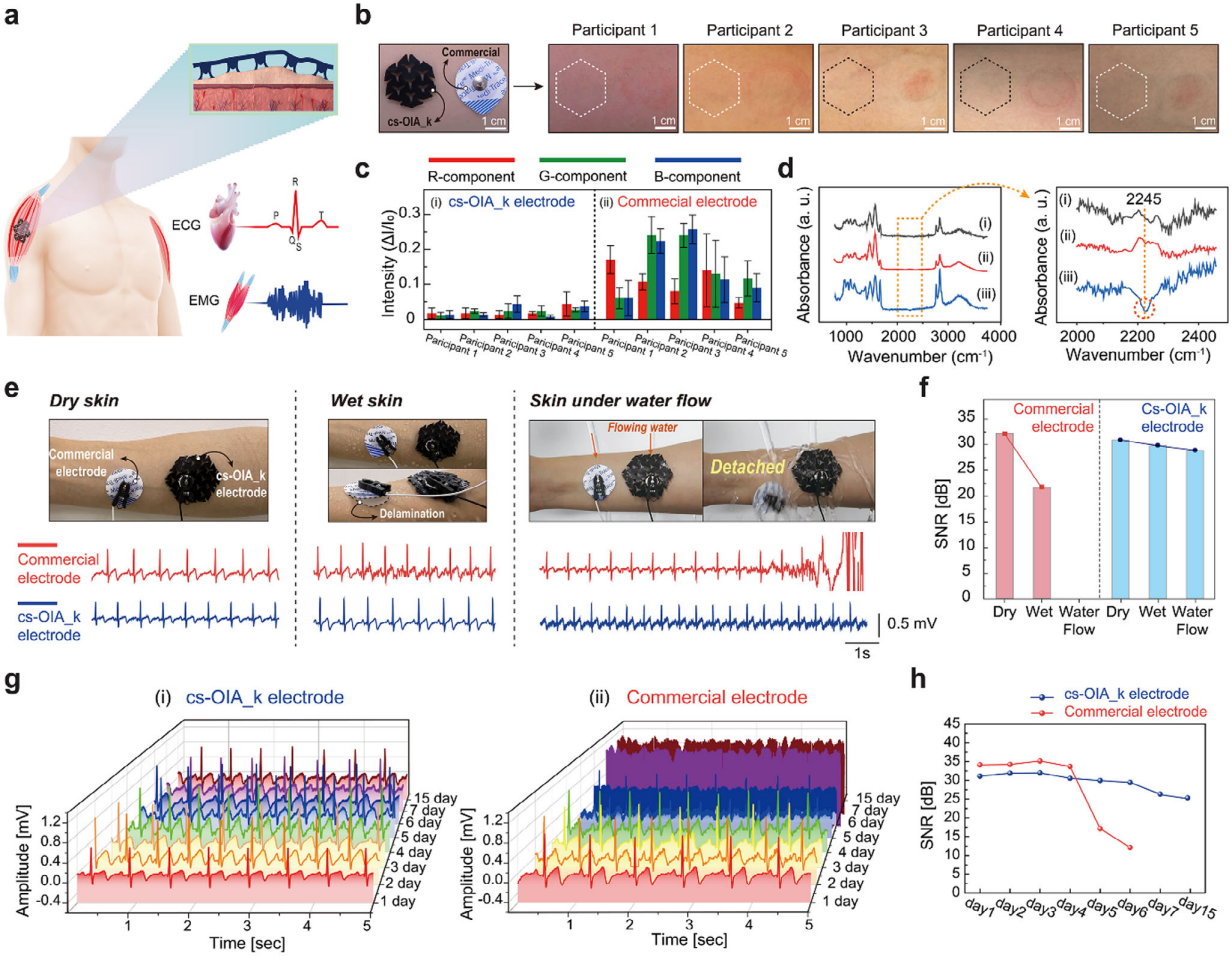
Comparison of cs‐OIA_k electrode performance with commercial electrodes in various skin conditions. a) Schematic illustration of the Cs‐OIA_k electrode applied for ECG and EMG signal monitoring b,c) Skin irritation evaluation of commercial electrodes and cs‐OIA_k. Error bars represent standard deviations (n = 5). d) FTIR spectra of the porcine skin surface i) when cs‐OIA_k was used ii) and when chemical adhesive iii) was used. Here, surface spectra (ii and iii) were obtained from the liver surface after separating the composite organ adhesive and chemical adhesive. e) ECG monitoring using commercial and cs‐OIA_k electrodes in dry, wet, and dynamic water flow conditions. f) SNR analysis of commercial and cs‐OIA_k electrodes in dry, wet, and dynamic water flow conditions. Error bars represent standard deviations (n = 5). g) long‐term ECG monitoring over a 15‐day period using by i) the Cs‐OIA_k electrode, and ii) the commercial electrode. h) SNR analysis over time for the commercial electrode and the Cs‐OIA_k electrode. Error bars represent standard deviations (n = 5).

Electrocardiogram (ECG) and electromyogram (EMG) signals, which are crucial vital signs, were measured using a cs‐OIA_k patch comprising conductive materials attached to the skin, which demonstrated the application of the cs‐OIA_k electrode for bio‐signal monitoring (**Figure** **5**a; Figure S19, Supporting Information). The changes in the skin surface were observed when both our adhesive device and commercial electrodes were attached and removed after 6 h (Figure 5b), to evaluate potential skin damage caused by the electrodes with five participants. Unlike the cs‐OIA electrode, which blocked droplet passage, the cs‐OIA_k electrode exhibited high liquid permeability, facilitating easy liquid passage (Figure S20 and Movie S6 Supporting Information). Due to its high permeability, the cs‐OIA_k electrode achieved clean adhesion without side effects, whereas the commercial electrode caused reactions such as itching, stickiness, and redness in the affected area. The hypoallergenic properties of cs‐OIA_k were confirmed through a quantitative evaluation of skin color changes (normalized intensity: ΔI/I_0_ before and after adhesive removal) (Figure 5c). In this analysis, the skin color change intensity ranged from ≈0.05 to 0.3 for the commercial electrode and 0 to 0.5 for the cs‐OIA_k, indicating the enhanced comfort and suitability of the cs‐OIA_k electrode for long‐term use. Additionally, FTIR spectra showed reduced peaks associated with irritants for the cs‐OIA_k electrode, further confirming its superior compatibility with biological tissues (Figure 5d). The cs‐OIA_k electrode demonstrated consistent adhesion and stable signal quality (SNR ≈29–30 dB) across various skin conditions (dry, wet, and under water flow). In contrast, the Ag/AgCl electrode exhibited notable signal reduction (SNR ≈15–20 dB) along with frequent detachment (Figure 5e,f). In particular, the cs‐OIA_k electrode with high adhesion strength under wet and rough skin resisted delamination from the wet skin of the participants against flowing water and exhibited stable in‐situ ECG signals (Figure S21; Movie S7, Supporting Information). In contrast, the commercial electrode detached within 12 s at an equivalent flow rate (0.085 L s^−1^), leading to signal loss. Unlike Ag/AgCl electrodes, which detach within 5 days due to material limitations, the cs‐OIA_k electrode maintained adhesion and signal stability for up to 15 days, demonstrating its potential for long‐term biomonitoring (Figure 5g,h). Furthermore, the cs‐OIA_k electrode demonstrated robust adhesion and durability despite drastic environmental changes (e.g., increased humidity), while also confirming its excellent biocompatibility (Figure S22, Supporting Information).

### 2.6 Biosignal Monitoring under Conditions of Continuous Movement

**Figure 6:**
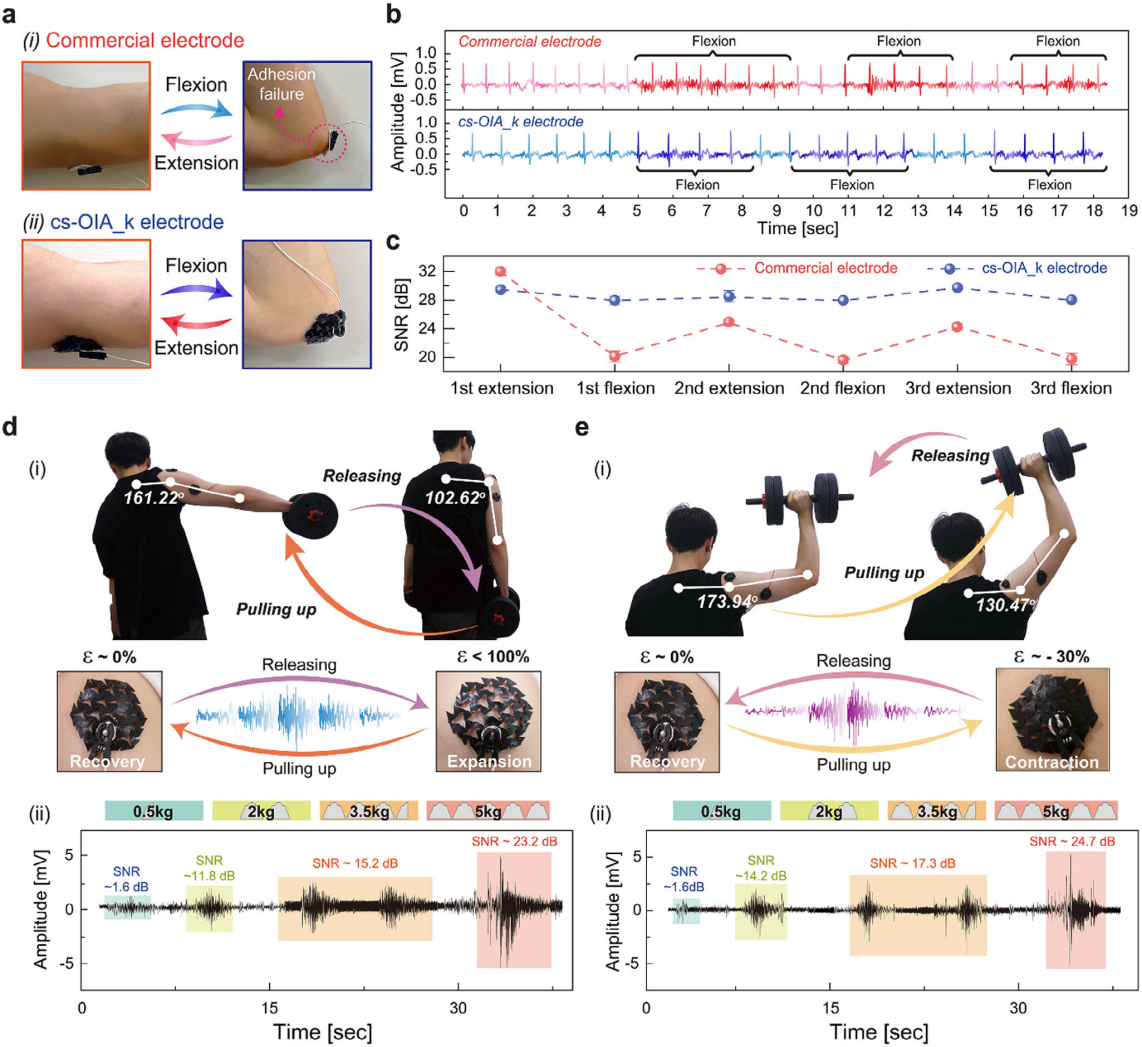
Highly adaptive permeable bio‐signal monitoring during dynamic exercise. a) Digital images of the adhesive stability of the commercial electrode and cs‐OIA_k electrode applied to the elbow. b) ECG signal monitoring of commercial electrodes and cs‐OIA_k during repeated bending cycles. c) SNR analysis of the Cs‐OIA_k and commercial electrodes during extension‐ cycles. Error bars represent standard deviations (n = 5). d,e) Application of body movements when cs‐OIA_k is attached to the curved part of the human body (shoulder, arm). d) i) image of an EMG test when cs‐OIA_k electrode is expanded. ii) EMG signal based on weightlifting movements. e) i) Image of an EMG test when the cs‐OIA_k electrode was contracted. ii) EMG signal based on weightlifting movements.

The robust adhesion of the cs‐OIA_k electrode was confirmed even under various dynamic body movements, ensuring stable biosignal acquisition (**Figure** **6**). Figure 6a–c illustrates ECG signal measurements using cs‐OIA_k electrodes attached to highly curved and wrinkled areas, such as the elbow. Despite repetitive flexion and extension motions of the arm, the cs‐OIA_k electrode maintained stable signal quality (SNR ≈25–30 dB), demonstrating its reliability during body movements. In contrast, commercial electrodes, which lack adaptability to skin deformation, experienced adhesion failure, leading to significant signal degradation (SNR ≈20–35 dB).

Figure 6d,e demonstrates stable EMG signals during body movements, causing continuous muscle expansion and contraction (≈−60° to 40° joint deformation), when the cs‐OIA_k electrode was attached to curved areas of the body, such as shoulder and arm. The cs‐OIA_k electrode with the kirigami pattern exhibited high adaptability to skin deformation (≈100% expansion and 30% contraction) and maintained stable and accurate EMG signal readings (SNR: 0–25 dB).^[^ ^46^ ^]^ EMG signal quality analysis further verifies measurement reliability by showing SNR improvement that is correlated with increased muscle load (Figure S23, Supporting Information). As shown in Figure 6d, a volunteer with cs‐OIA_k electrodes attached to his shoulder and arm performed dumbbell lifting exercises. During this activity, the joint angles of the volunteer deformed by ≈60°, and the cs‐OIA_k electrodes attached to the shoulders and arms repeatedly expanded (≈100%) and recovered in accordance with his movement. Furthermore, a steady increase in the EMG signals was observed as the volunteer lifted different weights (0.5, 2, 3.5, and 5 kg) (Figure 6d‐ii). Similarly, when the volunteer lifted and lowered the dumbbell (Figure 6e‐i), the joint angles deformed by ≈40°, resulting in skin contraction (≈30%) and recovery. The cs‐OIA_k electrodes exhibited high adaptability to these skin deformations as well, with the EMG signals increasing according to the weight and demonstrating different characteristics from those of the previous movements (Figure 6d). In contrast, the cs‐OIA electrode detached from the shoulder during the dumbbell lifting exercise, resulting in inconsistent measurements. (Figure S24, Supporting Information) This observation underscores the superior performance of the cs‐OIA_k, which maintains reliable electrical conductivity and adhesion under dynamic deformation. These findings highlight its potential for enabling stable and accurate bioelectronic applications, even on complex and highly mobile skin surfaces.

## 3 Conclusion

We proposed a super‐adaptive omni‐stretchable reversible kirigami‐based conductive adhesive patch that integrated a softened double‐layered octopus‐inspired suction cup structure with a kirigami‐based back plate. This design ensured strong adhesion and multi‐axial stretchability, even on curved, irregular, and wet skin surfaces. The conductive soft elastomer coated on the elastic microstructure maintained high conformance on both uneven and smooth surfaces, which enables stable long‐term monitoring of bio‐signals. In addition, the kirigami meta‐structure supporting the suction cups alleviated stress concentration, which resulted in stable adhesion on inflated curved surfaces without folding or detachment. Compared to conventional commercial electrodes, our permeable reversible adhesive electronics demonstrated superior practical applications in bio‐signal monitoring by reliably detecting EMG and ECG signals with minimal noise under previously challenging various conditions, including dry, sweaty, and constant joint movement, causing swelling and contraction of the skin due to muscle contraction. These results highlight the potential of the proposed multi‐axially stretchable electronics interface material for next‐generation human‐friendly super‐adaptive reversible bioelectronics. Furthermore, future developments could leverage 3D printing technology to streamline the fabrication process for complex 3D structures, further enhancing the system's applicability. Combining this approach with stimulus‐responsive materials (e.g., responsive to temperature, magnetic fields, or light) could open new avenues for intelligent materials in soft robotics and therapeutic skin interfaces, enabling the development of 4D‐printed adhesive actuators.^[^ ^47^ ^]^ Additionally, imparting biocompatibility and self‐healing properties would expand its potential applications to implantable bio‐robotic devices for advanced diagnostics and therapeutic interventions.

## 4 Experimental Section

### Glossary

This section briefly describes technical terms used. These include terms related to biological structures, structural design techniques, electrical properties, mechanical properties, and biosignal measurements .

### Prepared Conductive Precursor

Single‐walled carbon nanotubes (SWCNTs, Sigma‐Aldrich) with a high aspect ratio (on average, ≈4 nm in diameter, ≈400 µm long) were used to improve the electrical properties. SWCNTs were dispersed in P3HT in a selective solution (chloroform) and purified through a filtering process that involved centrifugation to obtain uniform electrical and mechanical properties. Ecoflex‐0030 (Smooth‐On, Inc.) was used as the matrix for the SWCNT–Ecoflex composites. For mixing, suspensions containing SWCNT, P3HT, and chloroform were subjected to vortex mixing (30 min) followed by ultrasonication (1 h). Thereafter, Ecoflex‐0030 (1:1 ratio of Ecoflex‐0030 A and B) was added to the ultrasonicated conductive suspension, and the mixture was vortexed for 30 min. The suspension was degassed in a vacuum desiccator for 2 h, followed by thermal treatment at 85 °C in a baking oven. For the conductive‐softened double‐layered structure, a softer elastomer (siloxane‐based MED‐6342; NuSil) than Ecoflex‐0030 was also prepared using the same method.

### Fabrication of the cs‐OIA_k Patch

To fabricate the octopus‐inspired pattern master, a 3D printed master mold was created using 3D computer‐aided design software (Autodesk Fusion 360, Autodesk Inc.). The mold was then treated in a 1% diluted self‐assembled monolayer (SAM) solution comprising trichloro(octadecyl)silane (ODTS) from Sigma–Aldrich Inc., with hexane as the solvent. This process was performed for 1 h at 25 °C, followed by overnight baking in an oven at 60 °C. The master mold was then employed for polymer molding using a soft silicon elastomer (Ecoflex‐0030, Smooth‐On, Inc.). The prepolymer and curing agent were combined in a 1:1 ratio and applied to the master mold. After degassing, the mixture was cured at room temperature for 4 h. Thereafter, the polymer was extracted from the mold and treated in oxygen plasma for 5 min to increase the surface energy. Furthermore, the patch surface was exposed to a SAM solution (tridecafluoro‐1,1,2,2‐tetrahydrooctyl)‐trichlorosilane (FOTCS) from Gelest Corporation (Morrisville, USA) for 1 h at 60 °C. An additional surface coating was applied after introducing the conductive precursor into the SAM‐treated mold, degassing, and curing at room temperature for 4 h to ensure the stable adhesion of the adhesive cup. A conductive softened material composed of MED‐6342 and SWCNTs was spin‐coated onto a glass substrate at 500 rpm for 1 min. OIA was selectively transferred exclusively to the adhesive cup tip by inking the glass substrate for 5 s without external pressure. Subsequently, they were cured at 135 °C for 1 h on a clean glass substrate.

### Expansion Test of the cs‐OIA_k Patch on the Balloon

To evaluate the multi‐directional adhesion stability of cs‐OIA_k on an expanding object, cs‐OIA_k and flat patches were affixed to balloons inflated with nitrogen gas for 3 s. Nitrogen gas was injected into the balloon at a constant flow rate of 11 cm^3^/s to induce inflation.

### LED Interconnected cs‐OIA_k under Multi‐Axial Strain

An open circuit was created by disconnecting the central part of the cs‐OIA_k patch. The LED and external wiring were connected to the cs‐OIA_k patch using conductive silver epoxy. The LED was activated by applying ≈3.0 V from a power supply. Subsequently, the patch was expanded in various directions, and the brightness and activation status of the LED were observed.

### Biosignal Measurement

A metal cap of a commercial ECG electrode (3M, Monitoring electrode) was superimposed on the cs‐OIA_k. ECG/EMG signals were measured under dry and sweaty conditions and were obtained from the electrodes connected to the ECG/EMG module (ADS1 × 9xECGFE, Texas Instruments). Three electrodes for ECG (negative, positive, and ground electrodes to the left wrist, left ankle, and right wrist, respectively) and three electrodes for EMG (two working electrodes to the arm and shoulder and a ground electrode on the arm) were affixed to the skin. Commercial electrodes prepared using the same protocol were used for comparison. Signal measurements of commercial electrodes and cs‐OIA_k were conducted independently. The experiments in human subjects were approved by the Institutional Review Board and were performed following the subject's informed consent (approval number: SKKU 2023‐11‐030).

## Conflict of Interest

The authors declare no conflict of interest.

## Author Contributions

G.R.K. and G.W.H. contributed equally to the study. G. R. K. performed conceptualization, methodology, investigation, visualization, wrote the original draft, wrote, reviewed, and edited. G. W. H. performed conceptualization, methodology, investigation, visualization, wrote the original draft, wrote, reviewed, and edited. D. L. and S. H. J. performed investigation. M. S. performed visualization C.‐H. H. performed investigation H. J. K. performed supervision, conceptualization, wrote, reviewed, and edited. C. P. performed supervision, conceptualization, methodology, wrote the original draft, wrote, reviewed, and edited.

## Supporting information



Supporting Information

Supplemental Movie 1

Supplemental Movie 2

Supplemental Movie 3

Supplemental Movie 4

Supplemental Movie 5

Supplemental Movie 6

Supplemental Movie 7

## Data Availability

The data that support the findings of this study are available from the corresponding author upon reasonable request.
