# Supplementary material for: Robustly Repeatable, Permeable, and Multi‐Axially Stretchable, Adhesive Bioelectronics With Super‐adaptive Conductive Suction Cups for Continuously Deformable Biosurfaces
Source: Adv Sci (Weinh). 2025 Mar 31;12(25):2500346. doi: 10.1002/advs.202500346 (PMC12224995; doi:10.1002/advs.202500346)
Supplement: Supplementary file 1 — Supporting Information [file ADVS-12-2500346-s006.docx]

Supporting Information

**Robustly Repeatable, Permeable, and Multi-Axially Stretchable, Adhesive Bioelectronics with Super-adaptive Conductive Suction Cups for Continuously Deformable Biosurfaces**

*Gyun Ro Kang^†^, Gui Won Hwang^†^, Dohyun Lim, Seung Hwan Jeon^,3^, Minwoo Song, Chan-Hwa Hong, Hye Jin Kim^*^, Changhyun Pang^*^*

G. R. Kang, G. W. Hwang, D. Lim, S. H. Jeon, M. Song, Prof. C. Pang

School of Chemical Engineering, Sungkyunkwan University (SKKU), 2066 Seobu-ro, Jangan-gu, Suwon, Gyeonggi-do 16419, Republic of Korea

C.-H. Hong, H. J. Kim

Electronics and Telecommunications Research Institute (ETRI), Daejeon 34129, Republic of Korea

S. H. Jeon

Mechanical Metrology Group, Korea Research Institute of Standards and Science, Daejeon 34113, Republic of Korea

Prof. C. Pang

Samsung Advanced Institute for Health Sciences and Technology (SAIHST), Sungkyunkwan University, Suwon, Gyunggi-do 16419, Republic of Korea

[^†^] G. R. K. and G. W. H. contributed equally to this work.

*Corresponding author. E-mail: nolawara@etri.re.kr (H.J.K.), chpang@skku.edu (C.P.).

**Keywords:** Bioelectronics, Wet adhesion, Bioadhesive, Kirigami, Composite.

**Supporting Experimental Section**

**Adhesion Test (Normal direction).** To measure the adhesion performance, we performed experiments in three directions (normal, shear, and peel-off directions). First, normal adhesion was measured against diverse substrates (glass, pig skin replica, porcine skin, and surfaces with varying roughness (Ra = 0, 40 µm, and 80 µm; area, approximately 3 × 3 cm^2^) at 25 °C under dry and wet conditions using custom-built equipment (adhesion tester, Neo-Plus, Korea). To measure under wet conditions, 300 μl of distilled water was dropped onto the substrate. All the adhesive measurement samples were manufactured with an area of 3 mm × 3 mm. As shown in Figure S9, the adhesive structure was fixed to the jig, and the target substrate was fixed to the bottom of the jig. The adhesive structure first touched the substrate using a preset preload. The repeated attachment and detachment processes were performed at least 10 times, and the adhesive force was obtained as the average of these values. Each test was performed at least five times.

**Adhesion Test (Shear and Peel-off direction).** The shear adhesive force was measured by first attaching the adhesive to the glass substrate and subsequently pulling it in the x-axis direction at a constant speed (~ 0.1 mm/s). Similarly, the average adhesive force was obtained by repeated measurements. To measure the peel-off adhesion, the adhesive was fixed to the jig and attached to the substrate. A constant detachment angle and speed (~1 mm/s) were maintained during the measurements. Each test was performed at least five times.

**FEM simulation.** We performed FEM simulations using commercial software (COMSOL Multiphysics version 6.2, license number 5094592, Altsoft, South Korea) to analyze the stress and material deformation rate applied during the detachment and deformation of the auxetic adhesives. We designed 3D models of the triaxial and flat adhesives, and meshes of these models with triangular elements were created automatically. The mesh sizes ranged from 0.38 mm to 0.93 mm. For simplicity, pseudo-static analysis and a linear elastic material model were used. The elastic modulus was set to 52 kPa, and Poisson’s ratio was set to 0.49 for this specific model.

**Supporting Theory Section**

**Derivation of the effective strain influencing the electrical conductivity of the kirigami structure.** The change in the electrical properties of the conductive nanocomposites was mainly caused by the change in the conduction path between the nanoparticles. Therefore, the effective strain influencing the electrical conductivity was analyzed to understand the electrical stability of the kirigami structure. First, the deformation mechanism owing to the tension of the 3D kirigami structure capable of geometric deformation was classified and organized into two states: (1) structural rearrangement was the main state, and (2) material deformation was the main state (see Figure. S8). Initially, when structural deformation was predominantly applied, the material strain of the kirigami structure was almost non-existent (State 1). After the structural rearrangement was completed, the material elongation began to increase as the material deformation was mainly applied (State 2). From this perspective, the total strain length ($\Delta l_{ts}$) of the 3D kirigami structure was equal to the sum of the structural rearrangement strain length ($\Delta l_{srs}$) and the material strain length ($\Delta l_{ms}$) and can be expressed as follows:

$\Delta l_{ts}=\Delta l_{srs}+\Delta l_{ms}$. (1)

Considering that the material strain started in state 2, each strain length was expressed through the initial length before deformation and the strain ratio.

$l_{i}\cdot\varepsilon_{ts}=l_{i}\cdot\varepsilon_{srs}+l_{im}\cdot\varepsilon_{ms}$, (2)

where $l_{i}$ is the initial length, $\varepsilon_{ts}$ is the total strain ratio, $\varepsilon_{srs}$ is the structural rearrangement strain ratio, $l_{im}$ is the initial material strain length, and $\varepsilon_{ms}$ is the material strain ratio. The effective strain influencing the electrical conductivity was minimally affected by the structural rearrangement and was significantly affected by the material strain. Consequently, when considering the material strain ratio based on the geometric structure of the auxetic structure, it can be expressed as

$\varepsilon_{ms}=\frac{l_{i}}{l_{im}}\left( 1+\varepsilon_{ts} \right)-1=2\frac{\sin\left( 2\theta-\frac{\pi}{6} \right)+\sin\left( \frac{\theta}{2}+\frac{\pi}{3} \right)}{2+\sqrt{3}}\left( 1+\varepsilon_{ts} \right)-1$, (3)

where $\theta$ is the included angle of the auxetic structure. Finally, the effective strain influencing the electrical conductivity that can infer the change in the conduction path is expressed as follows, considering the actual material tensile strength and the material-specific Poisson's ratio ($\nu$).

$\varepsilon_{esc}=\varepsilon_{ms}-\frac{\nu}{2}(\varepsilon_{ms}+\varepsilon_{ms}^{2})$ (4)

**Detailed derivation of the cs-OIA adhesion on dry substrate.**

**
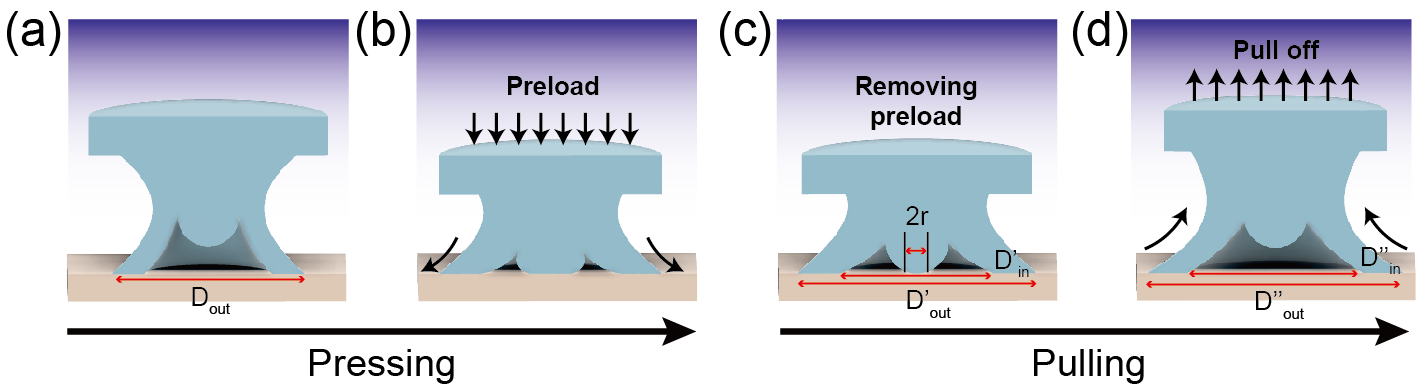
**

**Figure S1.** Adhesion process of the octopus-inspired flared suction cup. a, b) Pressing the suction cup for attachment and c, d) pulling for detachment.

The overall adhesion process of the cs-OIA can be divided into four parts, as shown in Figure S1: (1) Figure S1. (a) Initial contact state (2) S1. (b) Structural change under preload (3) S1. (c): equilibrium state of the elastic and adhesive forces when the preload was removed, and (4) S1. (d) cs-OIA structure during the separation process. The dry adhesive performance of cs-OIA first induces contact with the substrate and then undergoes structural changes through a preload that produces a suction-based attachment force. The dry adhesive stress ($\sigma_{t,dry}$), which is the suction-based attachment force of cs-OIA under dry conditions, can be expressed as the suction stress ($\sigma_{s,dry} \sim\Delta P\cdot A\cdot n)$ and the van der Waals stress ($\sigma_{vdw}\sim H_{c}\cdot A\cdot n/24h^{3})$ at the contact interface, as follows [1-3]:

$\sigma_{t,dry}=\sigma_{s,dry}+\sigma_{vdw}=(\Delta P\cdot(1-\gamma_{dry})\cdot A_{1}+\frac{H_{c}\cdot A_{2}}{24h^{3}})\cdot n$′″, (5)

where *ΔP* is the pressure difference between the pressure of the inner void dome and atmospheric pressure, $\gamma_{dry}$ is an experimentally determined constant between 0 and 1 that compensates for seal leakage at the cs-OIA regions of contact, *A_1_* _­_is the interfacial area of the suction effect, H_c_ is the Hamaker constant, *h* $\approx$0.165 nm is the cutoff distance, and n is the number of patterns. Since the air expands along with the change in volume of the inner cavity, *DP* is replaced by *P_0_* _·_*DV = P_0_* _·_ *(V*″*- V*′)*/V*″based on the ideal gas equation and Boyle’s law. *A_1_* and *A_2_* are described by $D_{in}^{''}$ , which is the inner diameter of the contact surface when a vacuum is established in the inner chamber, $D_{out}^{''}$ , which is the outer diameter of the contact surface when a vacuum is established in the inner chamber, and r is the radius of the contact protuberance. Therefore, the $\sigma_{t,dry}$ can be expressed as follows:
$\sigma_{t,dry}=\left[ \frac{{P_{0}(1-\gamma_{dry}){\pi D}_{in}^{''}}^{2}}{4}({\Delta V})+\frac{H_{c}\left( {D_{out}^{''}}^{2}-{D_{in}^{''}}^{2}+{4r}^{2} \right)}{24h^{3}} \right]\cdot n$, (6)

where *P_0_* is the atmospheric pressure, *V*′ is the inner void dome volume at the preload, and *V*″is the inner void dome volume at equilibrium in the absence of external forces and/or pulling.

**Detailed derivation of the cs-OIA adhesion on wet substrate.**

**
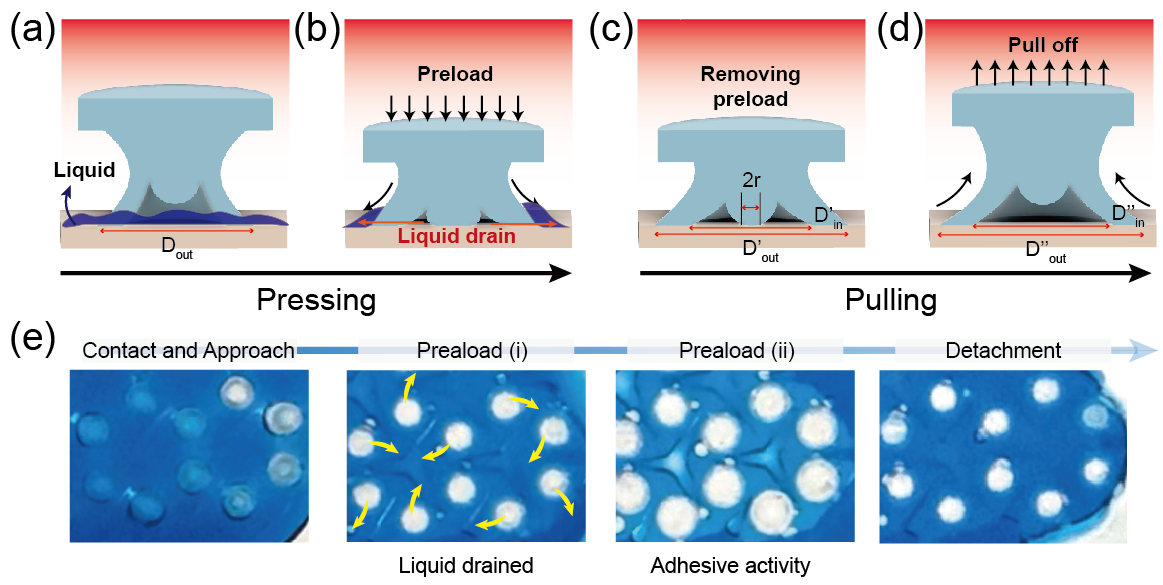
**

**Figure S2.** Adhesion process of the octopus-inspired flared suction cup under wet condition. a, b) Pressing the suction cup for attachment and c, d) pulling for detachment. e) OM image of water drainage during the adhesion process

In a wet environment, the total adhesive stress ($\sigma_{t,wet})$ was obtained by calculating the simple volume change upon deformation, interfacial area, and actual contact area. Since the water in the cs-OIA does not expand despite the internal volume change, the internal pressure can be assumed to be almost zero when it changes in a wet environment. Because the inner pressure is zero, the pressure difference between the interior and exterior of the cs-OIA can be maximized, which is expressed as ${\Delta P}_{max}=P_{o}$. Therefore, the suction stress of cs-OIA under wet conditions is expressed simply as ($\sigma_{s,dry}$ ~ ${P_{0}\cdot{\pi\cdot D}_{in}^{''}}^{2}/4$). The total adhesive stress of the cs-OIA structure in the wet state is defined by the summation of the suction stress ($\sigma_{s,wet})$ and capillary stress ($\sigma_{c}$ ~ $(F_{Laplace Pressure}+F_{Surface tension})/A_{sample}$). $F_{Laplace Pressure}$*/*$A_{sample}$ is the adhesive force generated at the contact interface, and $F_{Surface tension}$*_/_*$A_{sample}$ is the force generated around the contact interface in a wet environment. Therefore, the total adhesive stress can be expressed as follows:

$\sigma_{t,wet}=\left[ (1-\gamma_{w})\frac{{P_{0}{\pi D}_{in}^{''}}^{2}}{4}+\frac{\pi\cdot{{(D}_{out}^{''}}^{2}-{D_{in}^{''}}^{2}+4r^{2})}{4}\cdot\gamma\left( \frac{\cos\vartheta_{1}+\cos\vartheta_{2}}{h} \right)+l\cdot\gamma\right]\cdot n$, (7)

where *γ_w_* is a compensation factor that has a value between 0 and 1 owing to seal leakage at the cs-OIA regions of contact; *γ* is the surface tension of water; *θ_1_* and *θ_2_* are the contact angles on the adhesive and engaged substrate, respectively; *h* is the liquid gap; and *l* is the outer circumference.


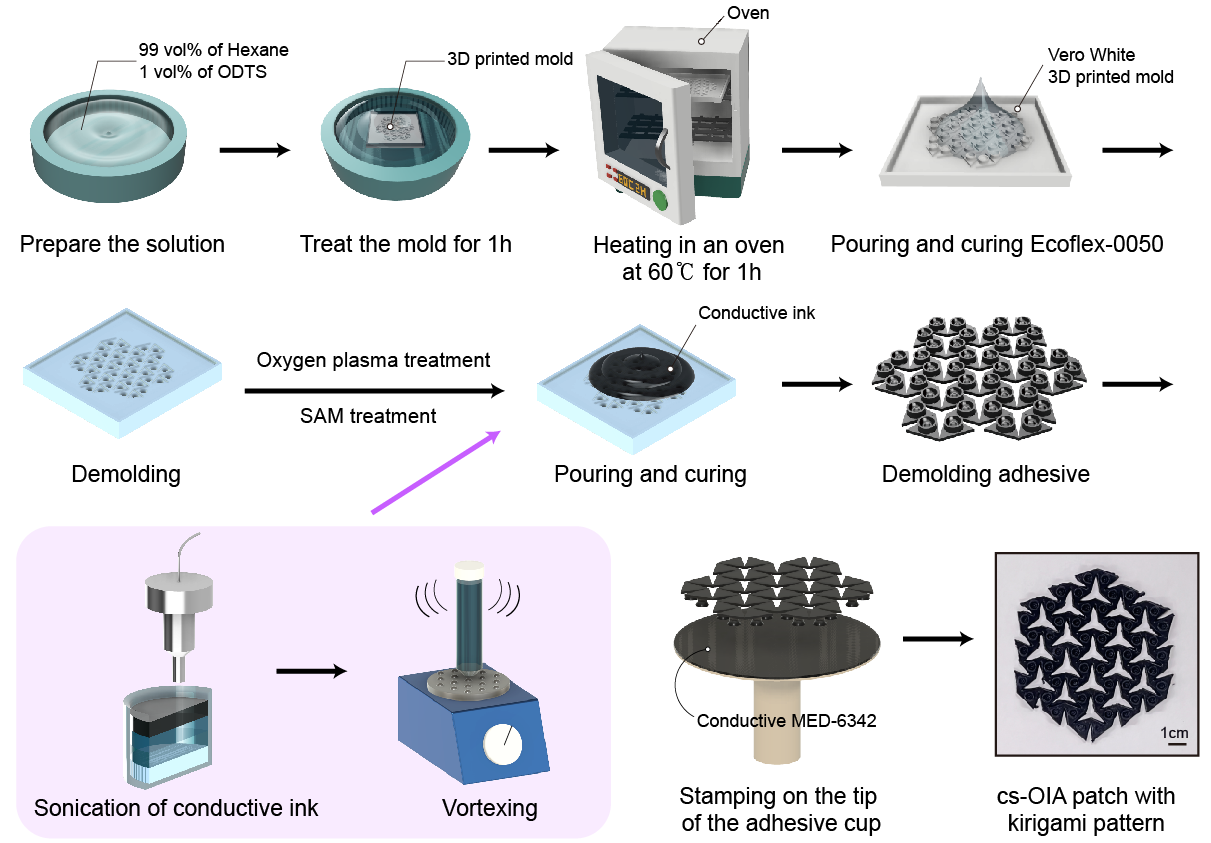


**Figure S3.** Fabrication of the conductive softened-layered octopus-inspired adhesive.


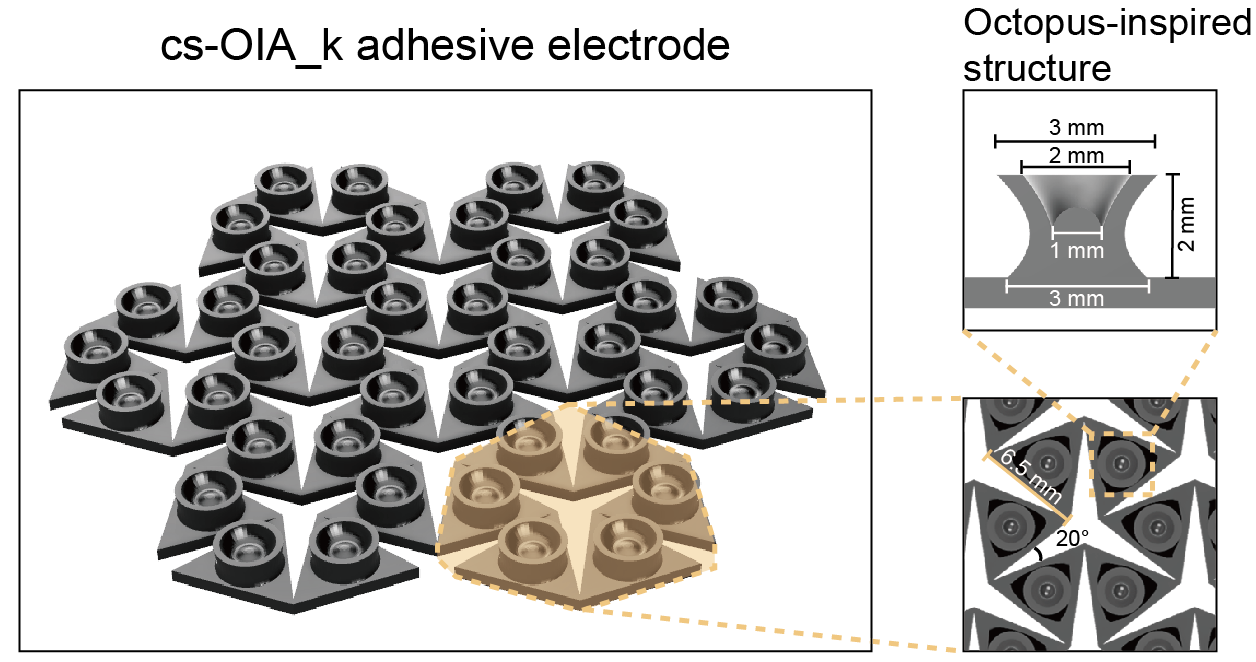


**Figure S4.** Detailed scale of the cs-OIA_k adhesive electrode.

**
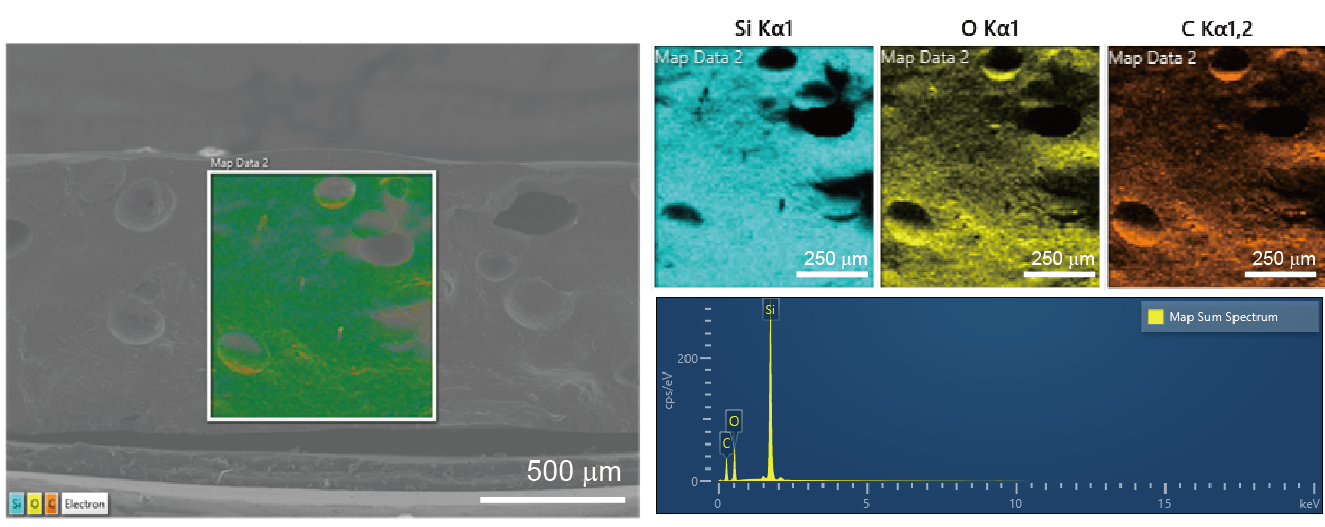
**

**Figure S5.** EDS spectrum of the SWCNT/Ecoflex-nanocomposites. Inset is the table showing the percentage of each component in the Ecoflex matrix.

**
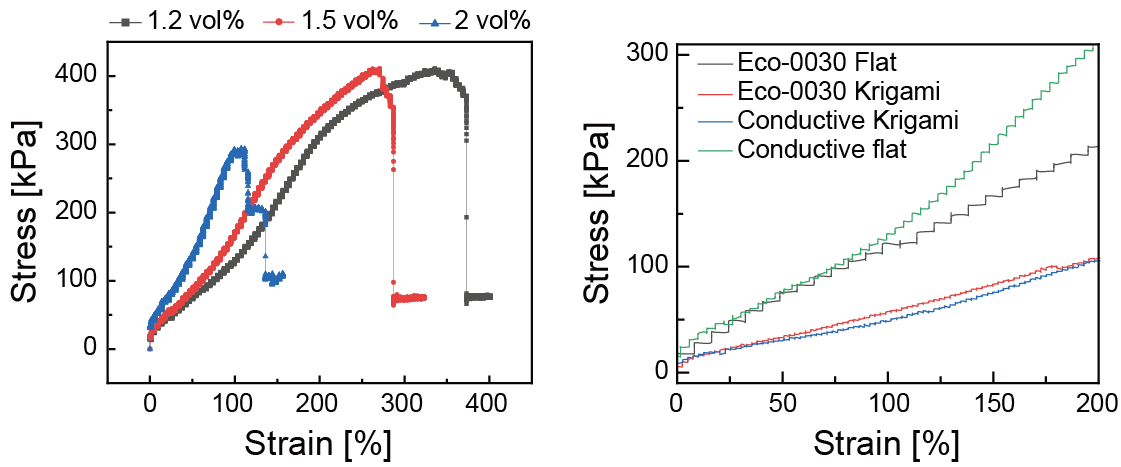
**

**Figure S6.** Stress–strain (S-S) curves of the flat composites according to the vol % of SWCNTs in the matrix.

**
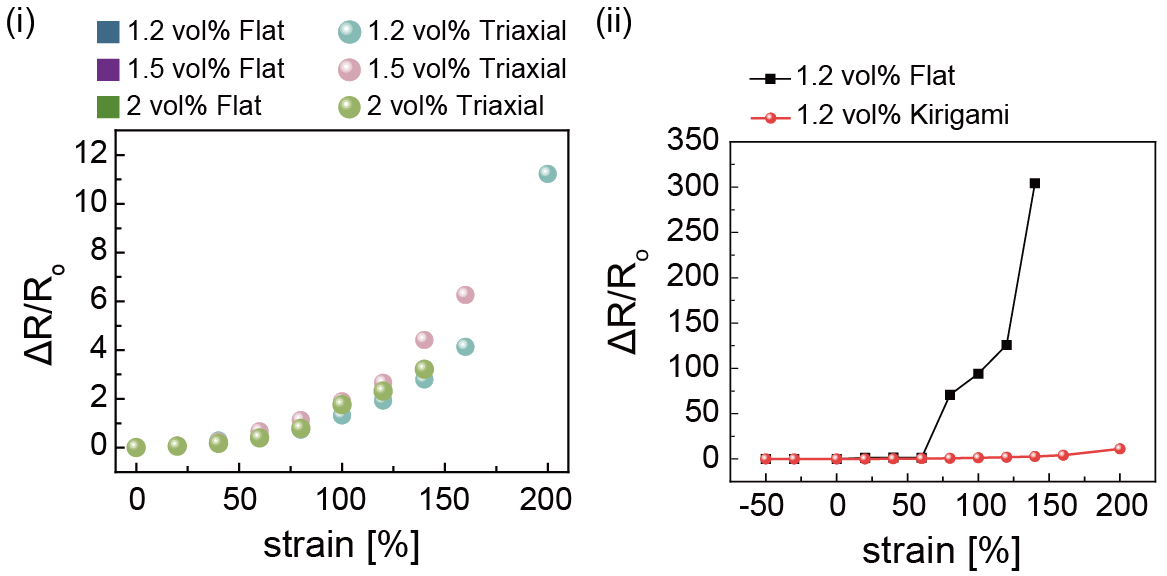
**

**Figure S7.** The electrical properties of the cs-OIA were represented by the rate of resistance change with applied strain, according to the composite concentration and structure. i) applied strain: 0~200%, ii) applied strain: -50~200%.

**
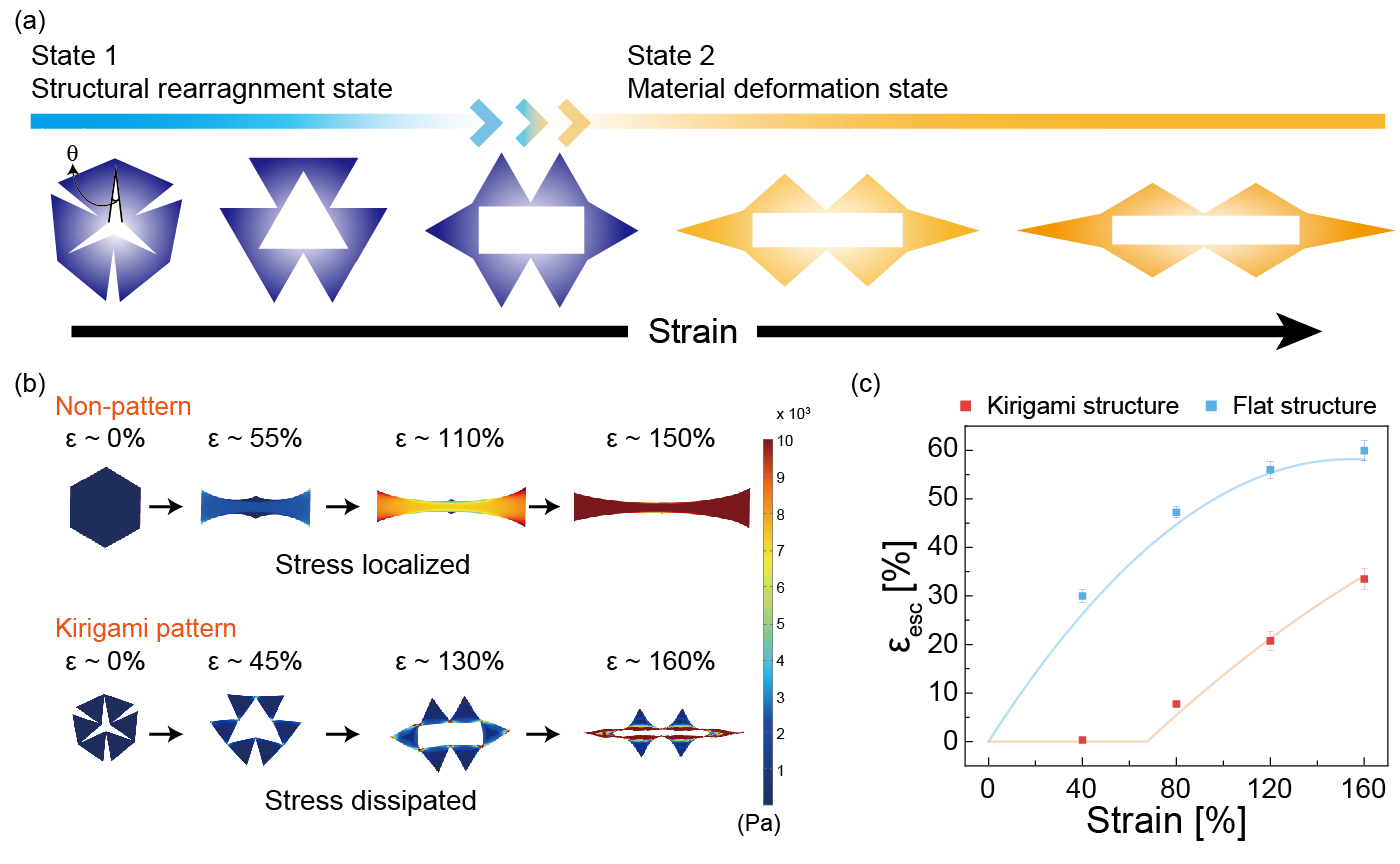
Figure S8.** a) State separation that resulted from structural rearrangement and material deformation under strain. b) Stress simulation of non-pattern and auxetic pattern under tensile strain. c) Comparison of the effective strain influencing the electrical conductivity under structural strain of the kirigami and flat structures.

**
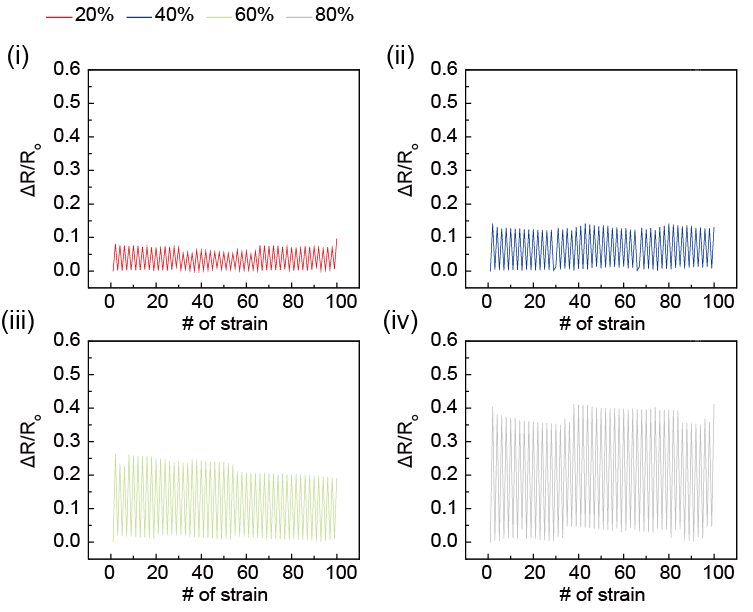
**

**Figure S9.** Cyclic measurements of electrical signals of the 1.2 vol% triaxial structure under 100 stretching (each strain %) cycles.

**
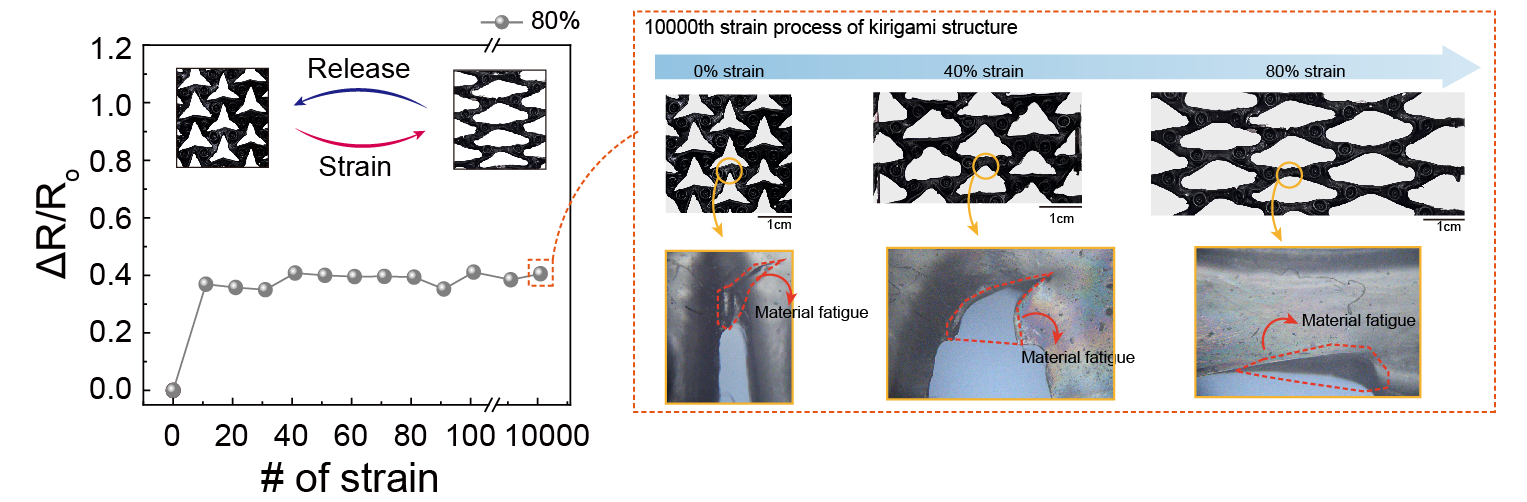
**

**Figure S10.** Electrical resistance changes and OM images of material fragile according to 10,000 repeated uses of cs-OIA_k**.**

**
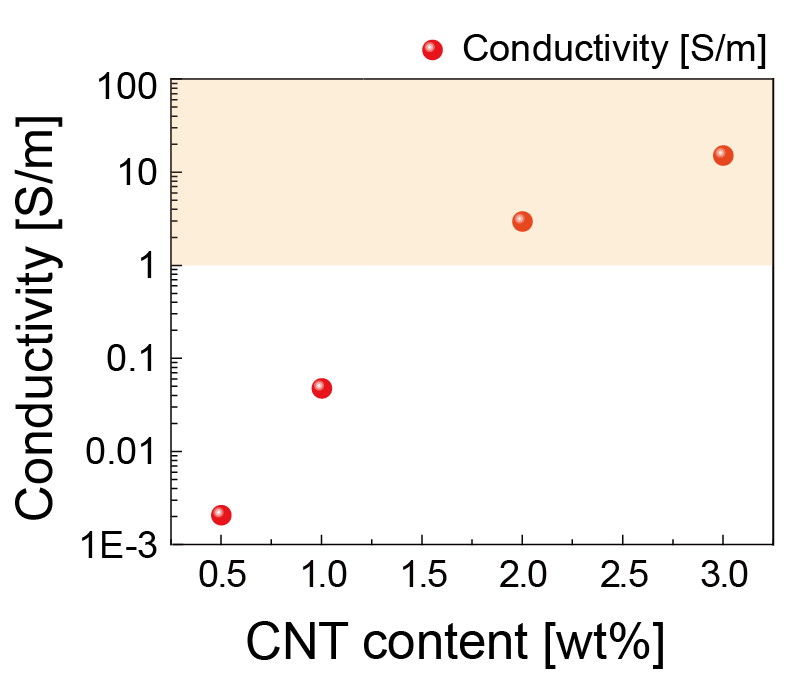
**

**Figure S11.** Conductivity of the soft elastomer composite based on the weight fraction of SWCNTs.

**
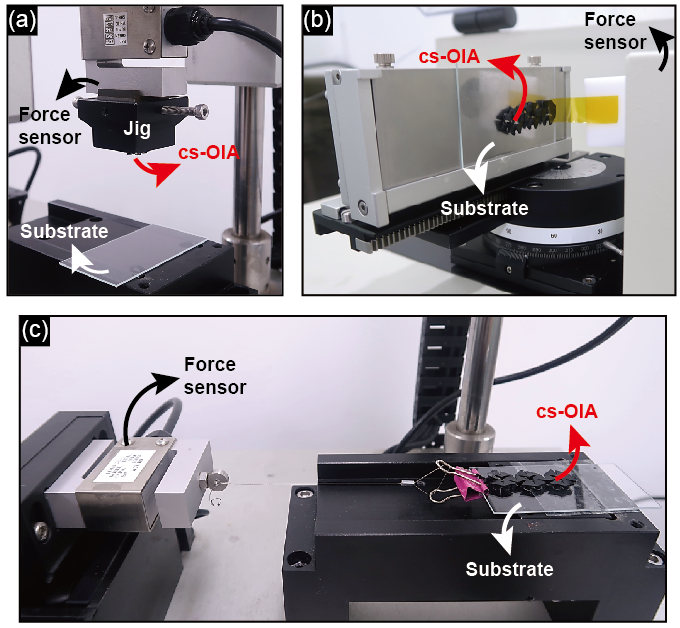
**

**Figure S12.** Measurements of adhesion on dry/wet substrates using a custom-built equipment. The adhesion strength was measured in various directions; a) Normal direction, b) Peel-off direction, c) Shear direction.

**
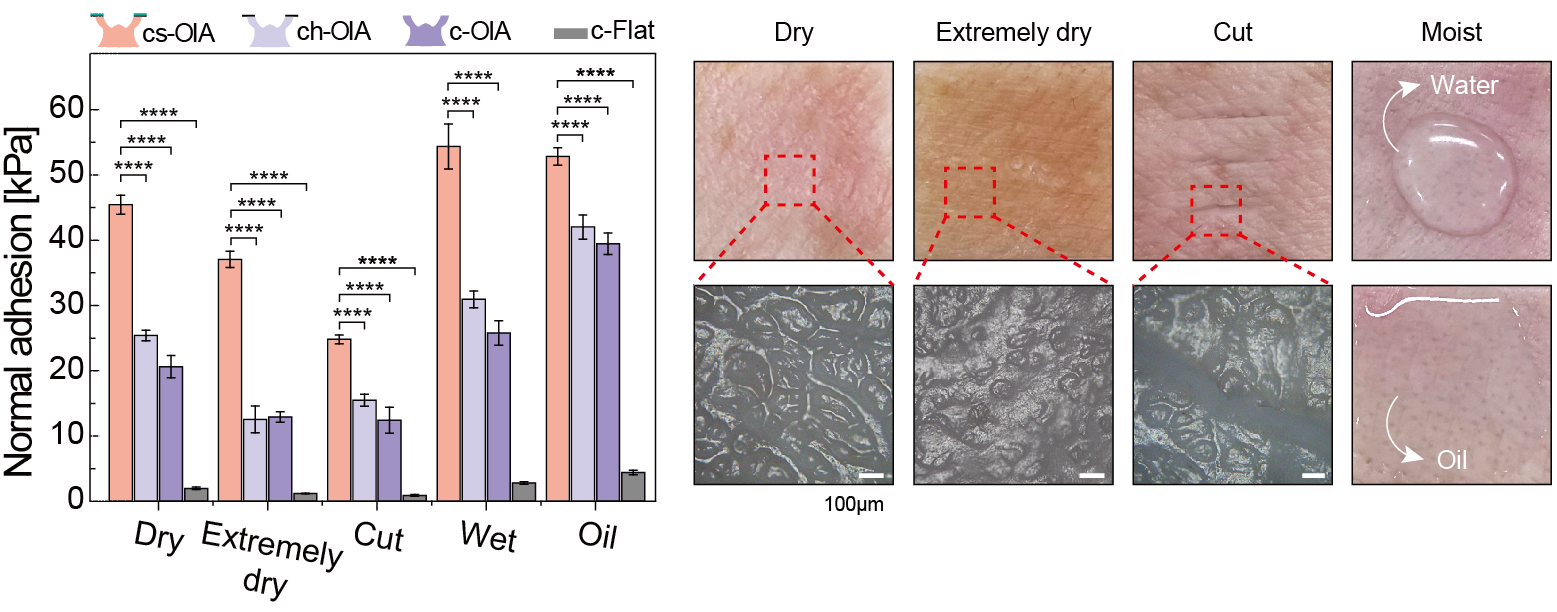
**

**Figure S13.** Adhesion performance of cs-OIA_k and comparison groups on various types of porcine surface. Error bars represent standard deviations (n = 5) Statistical significance was calculated: *p < 0.05; **p < 0.01; ***p < 0.001; ****p < 0.0001.


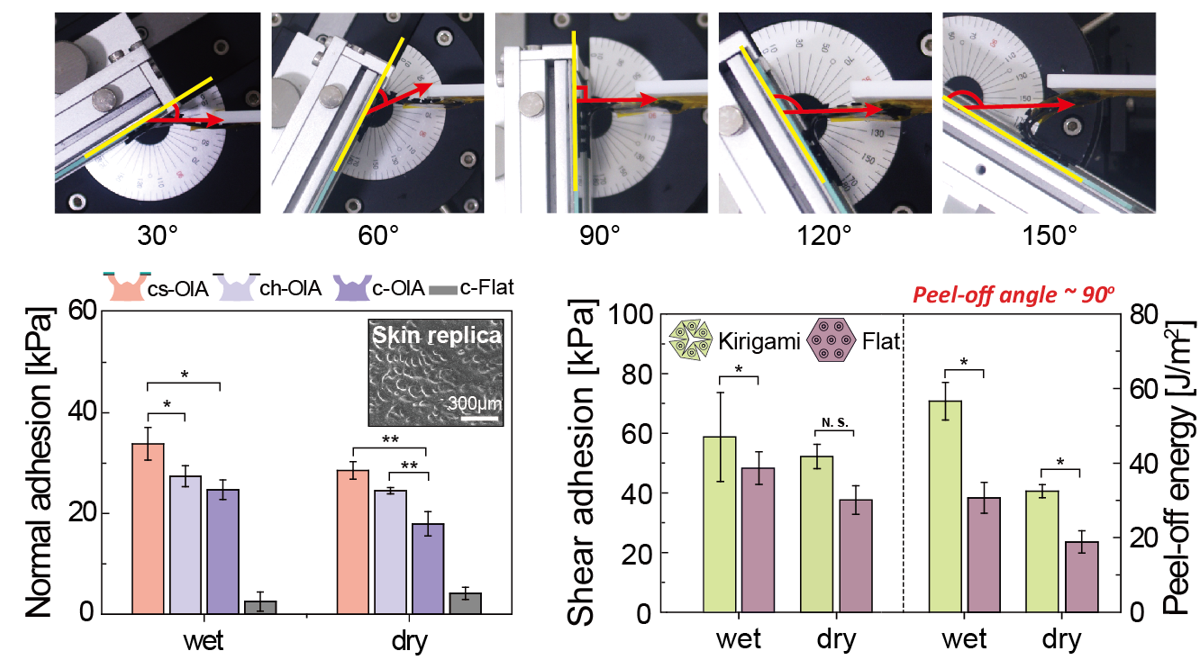


**Figure S14.** Normal and shear adhesion performance of the bioinspired adhesive architecture with kirigami pattern against pig skin replica under dry and wet conditions. Error bars represent standard deviations (n = 5) Statistical significance was calculated via t-test: *p < 0.05; **p < 0.01; ***p < 0.001; ****p < 0.0001.


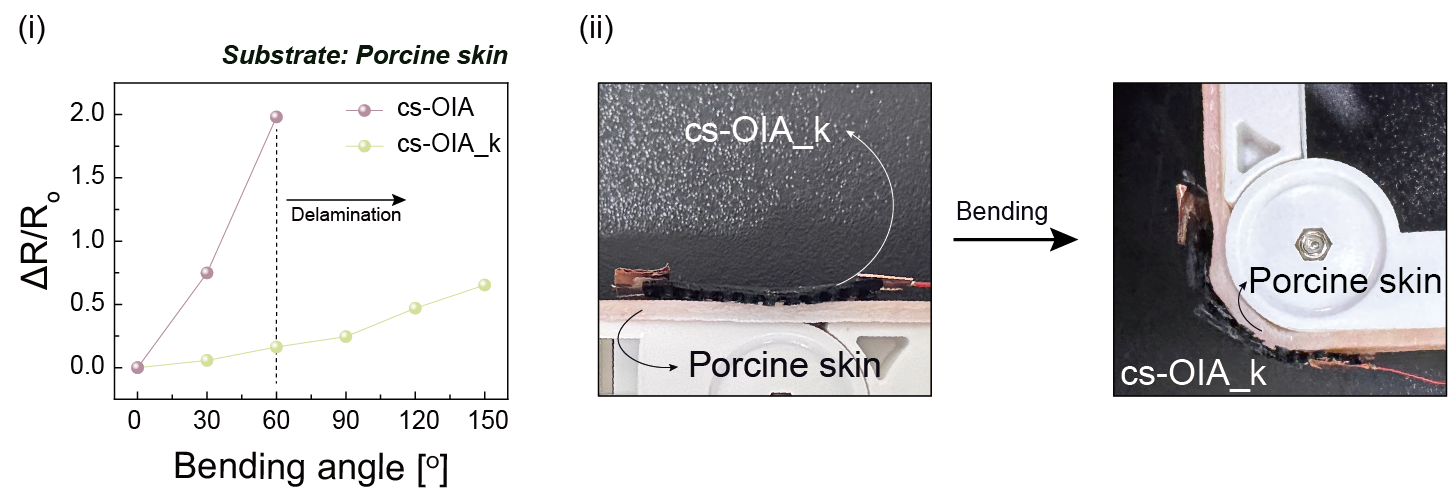


**Figure S15.** Measurement of adhesion retention performance and electrical resistance change of cs-OIA and cs-OIA_k on bent porcine skin. Error bars represent standard deviations (n = 5)


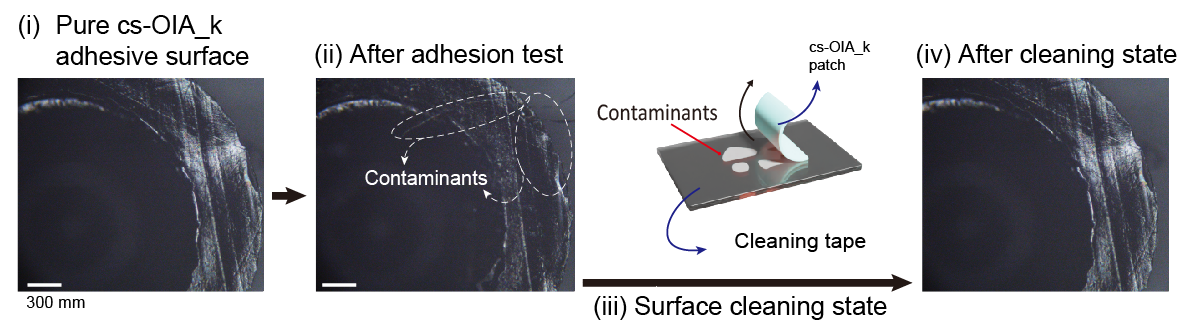


**Figure S16.** OM images of the adhesive surface of cs-OIA. i) Initial surface, ii) contaminated surface after 10,000 adhesion repetitions iii) Clean surface after surface cleaning.

**
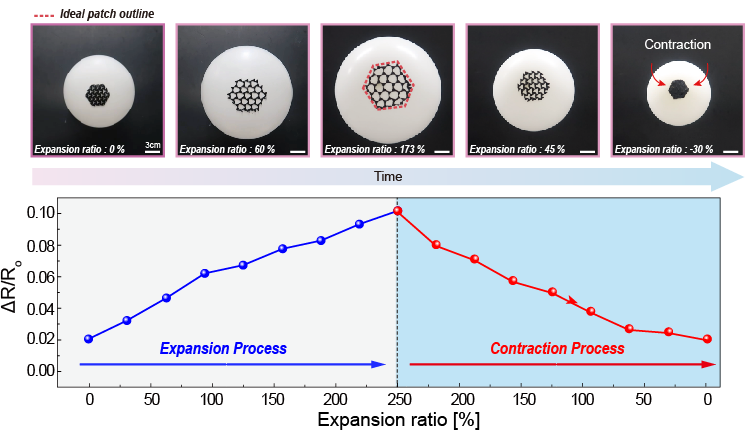
**

**Figure S17.** Snapshots of cs-OIA_k on a curved balloon that expanded up to 173% over time and then contracted to -30%. Electrical characteristics of cs-OIA_k as a function of balloon volume.

**
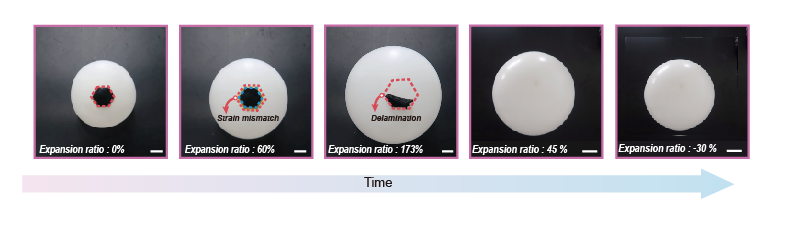
 Figure S18.** Snapshots of cs-OIA on a curved balloon that expanded up to 173% over time and then contracted to -30%.

**
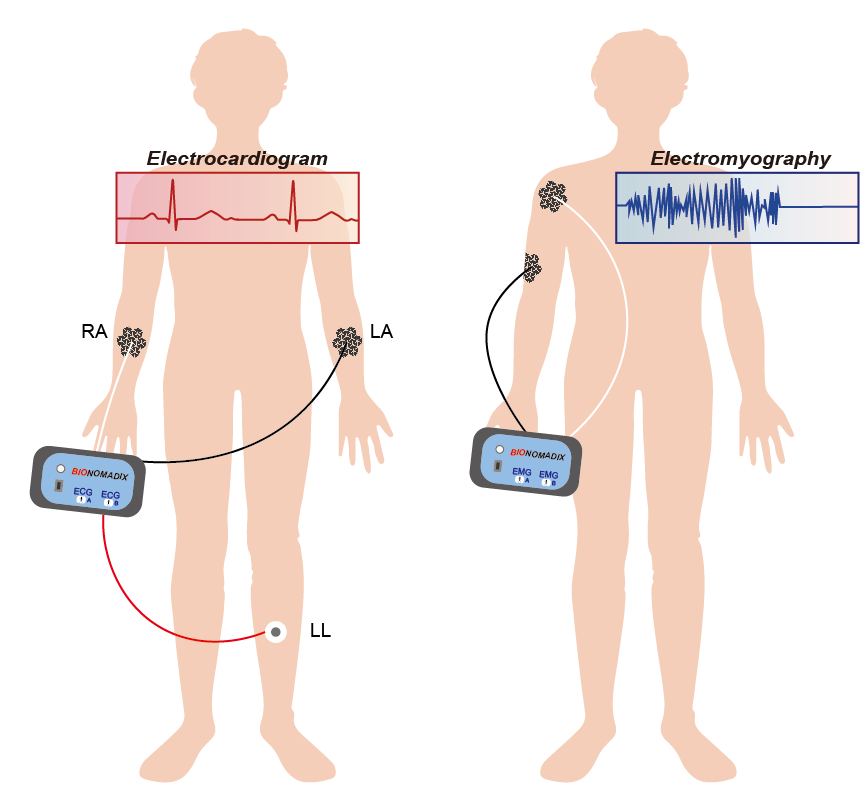
**

**Figure S19.** Schematic of cs-OIA_k adhesive electrodes to measure ECG and EMG signals.

**
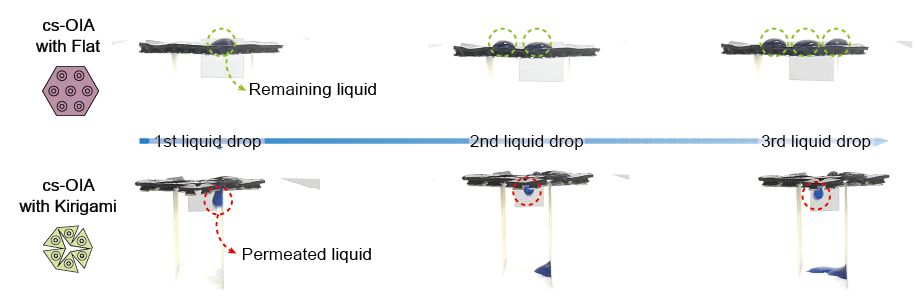
**

**Figure S20.** OM images of water permeation of cs-OIA and cs-OIA_k.


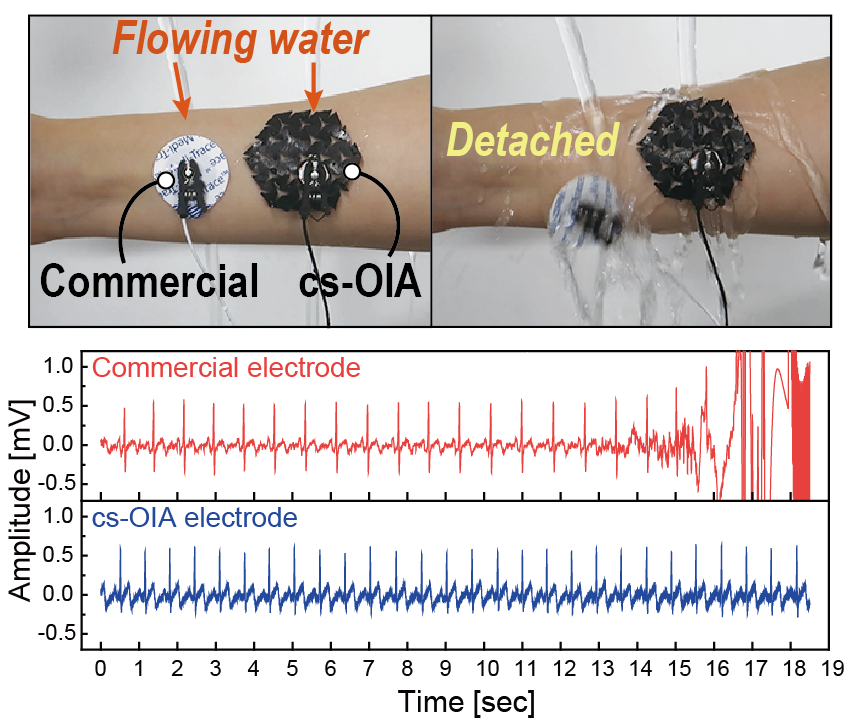


**Figure S21.** Testing the flow tolerance of the skin adhesive and commercial electrode in flowing water and demonstration of an cs-OIA_k electrode attached to human skin and the ECG signal measurement on sweaty skin in flowing water.


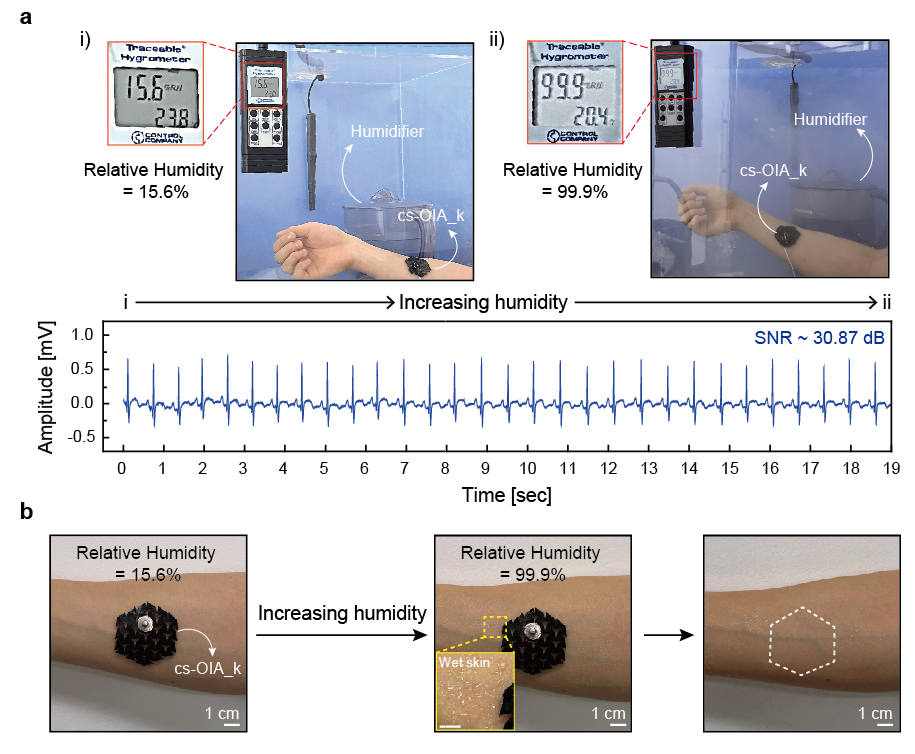


**Figure S22.** ECG monitoring and skin irritation measurment in extreme humidity environments with cs-OIA_k. a) i) Initial conditions : 15.6% humidity condition ii) Later condition: 99.9% humidity condition. b) Skin irritation evaluation of cs-OIA_k after in extreme humidity conditions.


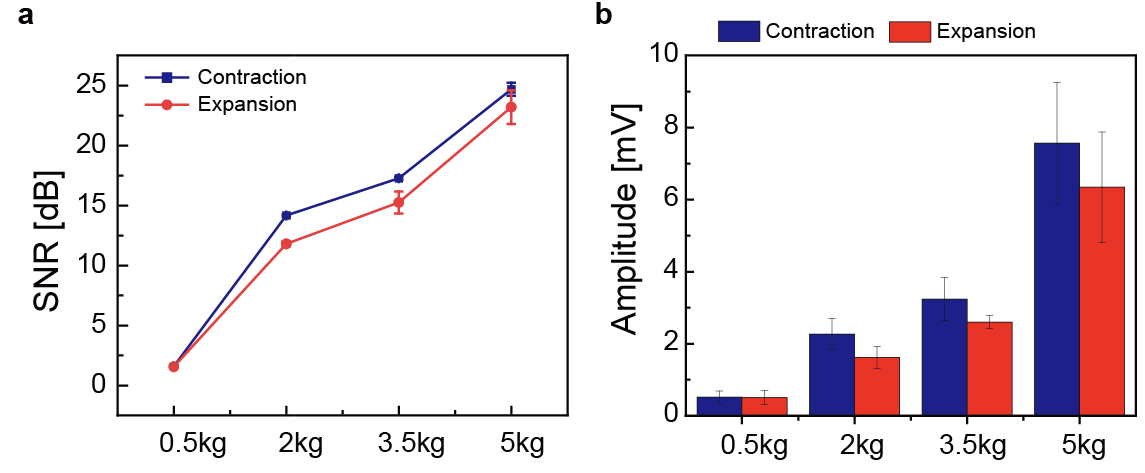


**Figure S23.** Analysis of amplitude and SNR in weight-dependent EMG signals obtained using cs-OIA_k.

**
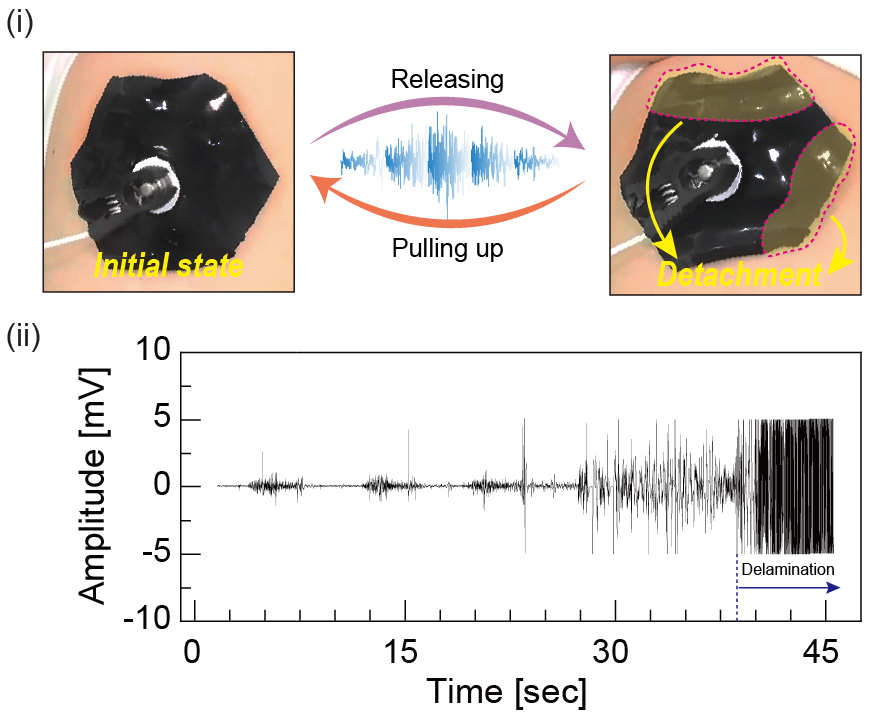
**

**Figure S24.** EMG measurement using cs-OIA electrodes attached to the shoulder and arm (i) image of an EMG test when cs-OIA electrode is expanded. (ii) EMG signal based on weightlifting movements.

**References**

1. Wang, S.*, et al.*, *Advanced Functional Materials* (2021) 31 (9), 2009217
2. Baik, S.*, et al.*, *Nature* (2017) 546 (7658), 396
3. Lee, J.*, et al.*, *ACS nano* (2024) 18 (7), 5311
